# Arginine Residues Modulate the Membrane Interactions of pHLIP Peptides

**DOI:** 10.1021/acs.jcim.3c00360

**Published:** 2023-07-03

**Authors:** Tomás F. D. Silva, Hannah Visca, Craig Klumpp, Oleg A. Andreev, Yana K. Reshetnyak, Miguel Machuqueiro

**Affiliations:** † BioISI—Instituto de Biossistemas e Ciências Integrativas, Faculdade de Ciências, Universidade de Lisboa, 1749-016 Lisboa, Portugal; ‡ Scuola Internazionale Superiore di Studi Avanzati, 34136 Trieste, Italy; ¶ Physics Department, University of Rhode Island, Kingston, Rhode Island 02881 United States

## Abstract

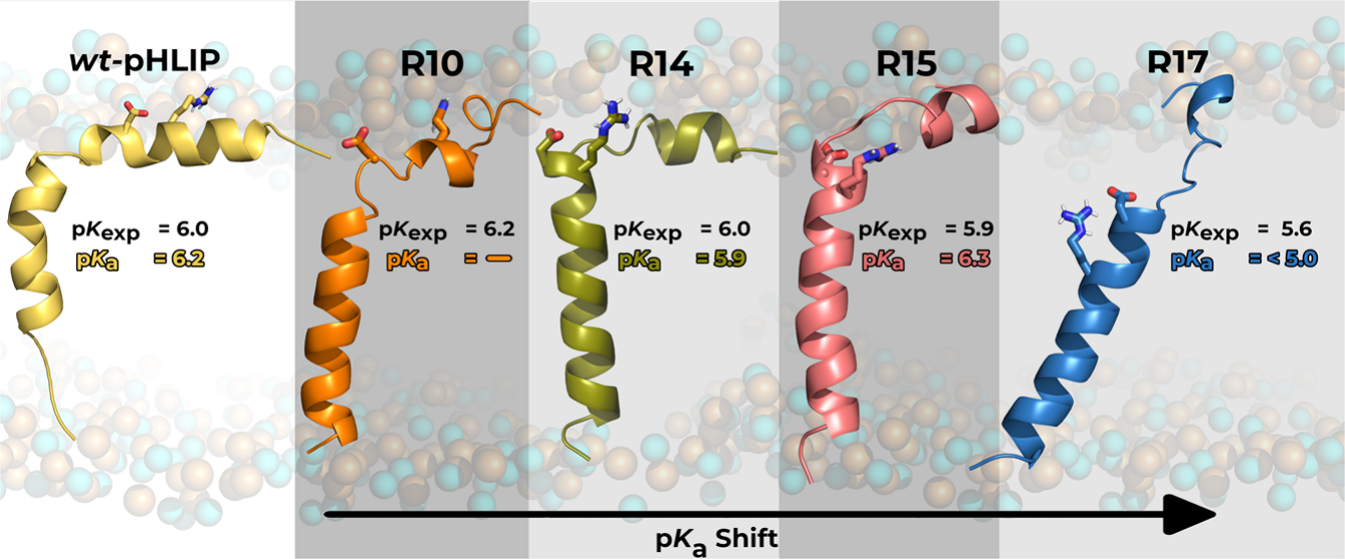

Most processes at the water–membrane interface often involve protonation events in proteins or peptides that trigger important biological functions and events. This is the working principle behind the pHLIP peptide technology. A key titrating aspartate (Asp14 in *wt*) is required to protonate to induce the insertion process, increase its thermodynamic stability when membrane-embedded, and trigger the peptide’s overall clinical functionality. At the core of pHLIP properties, the aspartate p*K*_a_ and protonation are a consequence of the residue side chain sensing the changing surrounding environment. In this work, we characterized how the microenvironment of the key aspartate residue (Asp13 in the investigated pHLIP variants) can be modulated by a simple point mutation of a cationic residue (ArgX) at distinct sequence positions (R10, R14, R15, and R17). We carried out a multidisciplinary study using pHRE simulations and experimental measurements. Fluorescence and circular dichroism measurements were carried out to establish the stability of pHLIP variants in state III and establish the kinetics of the insertion and exit of the peptide from the membrane. We estimated the contribution of the arginine to the local electrostatic microenvironment, which promotes or hinders other electrostatic players from coexisting in the Asp interaction shell. Our data indicate that the stability and kinetics of the peptide insertion and exit from the membrane are altered when Arg is topologically available for a direct salt-bridge formation with Asp13. Hence, the position of arginine contributes to fine-tuning the pH responses of pHLIP peptides, which finds wide applications in clinics.

## Introduction

The peptides and membrane interactions are vital for several biological processes, such as molecular transport, signaling pathways, and cell membrane integrity.^1−3^ While these processes are at the core of a wide array of research areas, the molecular interactions between protein and lipids are still hard to fully characterize in complex systems. Simple peptide models are widely popular as they are customizable; they mimic defining traits of membrane proteins and there are limitless combinations of peptides with membrane models to study different types of biological systems. The advent of these peptide models, such as GALA^4^ and WALP,^5^ propelled the study of transmembrane peptide design, from which were identified several residue sequence patterns that determine membrane folding and insertion, such as hydrophobic residue stretches (alanine and leucine) and outward hydrophilic residues (lysines, arginines, aspartates, glutamates).^6^ Several computational studies have focused on simple models, using single lipid membranes, to modulate and understand the physical chemistry of peptide–membrane interactions. These studies delved into modifying the peptide length, charged residues, and hydrophobic stretches to provide molecular insight into a wide range of biophysical phenomena, including peptide structural disposition in the membrane^7,8^ and the formation of membrane pores.^9,10^ Although most of these studies do not fully mimic the physiological environment, they provide important information to determine possible folding pathways^11,12^ and identify key residues for peptide function.^13−15^ Still, in other experimental studies, complex lipid compositions (i.e., cholesterol and anionic lipids) are often used in tandem with different ion concentrations to highlight how peptide kinetics changes as a result of the more electrostatically charged environments on the protonation changes of the relevant residues.^16−20^

One of the more clinically relevant peptide models is the pH-low insertion peptide (pHLIP), which can target imaging and therapeutic agents to tumors. The pHLIP family is characterized by long (28–40 amino acids) membrane-inserting peptides, whose distinguishing trait from other transmembrane model peptides is their acidity-dependent membrane insertion and folding.^21−26^*WT*-pHLIPs possess a kinked α-helical fold,^14^ more commonly occurring below pH 6.0 (state III). Otherwise, the peptide adopts a random coil conformation either adsorbed to the membrane surface (pH 7.0 to 8.0; state II) or in solution (pH > 8.0; state I).^21^ The pH dependency results from the titratable residues that populate the water–membrane interface. By fluctuating between the phosphate region and the deeper ester region, one of the key residues (Asp14) undergoes (de)protonation events, which either promote peptide insertion or membrane exiting.^14^ The proton binding affinity is a measurement of the energy needed to protonate a given residue and various factors affect this property: peptide movement, (un)folding, and intermolecular (membrane lipids) and intramolecular (side chains) interactions.^8,15^ While Asp14 mostly contributes to the stability of the inserted state, other titratable anionic residues play an important role in the kinetics of transitions between states.^14^ In our previous work, we characterized and identified the electrostatic interactions dictating the p*K*_a_ of *wt*-pHLIP^8,14^ and the Var3 peptide,^24,27^ which is in clinical trials with imaging and therapeutic agents, in a simple liposomal-like model and in a cell-like model that accounts for the existence of the pH gradient setup.^28^ We highlighted the necessity of including the pH gradient to accurately assess the therapeutic potential of transmembrane peptide models and we also described the intramolecular interactions that predetermine the peptide’s thermodynamic membrane stability. One of these fundamental interactions occurs between the key aspartate and a neighboring arginine residue. This creates a distinct aspartate electrostatic microenvironment, which impacts the residue proton binding affinity. The interactions of the arginine with both the aspartate and lipid headgroups were previously discussed as well.^15^

For many years, the role of cationic residues has been discussed in the context of studies about cell-penetrating peptides studies. A critical impact in inducing/hindering peptide insertion through morphological membrane alterations was established^29,30^ while also affecting the peptide structure and position within the membrane,^31^ including the possibility of promoting pore formations and membrane permeability.^9,29^ These effects hinge on the cationic residues’ behavior in the membrane, especially for arginines and lysines, as they remain positively charged as they move along the membrane normal.^32,33^ When an α-helical peptide is inserted into a membrane, the cationic residue is dragged from an energetically favorable solvent environment to an apolar lipid medium. Depending on the lipid bilayer region placement, the residue may interact with anionic (e.g., phosphate) groups, effectively working as a peptide anchor, or it can snorkel to minimize the energy cost associated with membrane embedding, pulling the peptide with it, as seen in KALP peptides.^34^

Several studies have strengthened the significance of cationic residues in transmembrane peptide models and their ability to modulate the peptide–membrane equilibrium. Furthermore, the presence of more charged groups near key peptide residues directly impacts the protonation and p*K*_a_ of such residues in pHLIP. In this study, we introduced and investigated several pHLIP Arg variants, based on a known single-Trp template,^25^ where the Arg residue was permuted at different distances and locations relative to the key Asp residue in a systematic manner and compared the experimental data with modeling calculations. We aim at characterizing and assessing the impact of these mutations in the context of transmembrane peptide design, while also discussing the impact on the peptide–membrane equilibria, folding stability, key residues p*K*_a_, and other relevant properties.

## Methods

### Synthesis of Peptides

pHLIP peptides were synthesized and purified by CSBio. The lyophilized peptides were dissolved in a buffer containing 2.7 M urea and then centrifuged through a G-10 column to remove urea. Concentrations of the peptides were calculated spectrophotometrically by measuring absorbance at 280 nm and using an extinction coefficient of 12,660 M^–1^ cm^–1^. At the concentration range used in the experiments, *wt*-pHLIP peptides are predominantly monomeric.^35,36^ The variants used here did not change significantly the overall number and type of amino acids, just their sequence. Therefore, it is unlikely to expect oligomerization of the investigated pHLIP variants at these concentrations.

### Fluorescence and Circular Dichroism (CD) Measurements

Using an excitation wavelength of 295 nm and 1 mm excitation and emission slits, tryptophan fluorescence spectra were recorded from 310–400 nm on a PC1 spectrofluorometer (ISS, Inc). The excitation polarizer was set to the magic angle, or 54.7°, while the emission polarizer was set to 0° to reduce Wood’s anomalies. CD spectra were recorded from 190–260 nm with 1 nm steps on a MOS-450 spectrometer (Bio-logic, Inc). The concentrations of the peptide and POPC were 7 μM and 1.4 mM, respectively, in each experiment. The temperature control was set to 298 K for both fluorescence and CD measurements.

The pH-dependent insertion of the peptides into the lipid bilayer of the POPC liposomes was studied by monitoring either the shift in the spectral maximum of the tryptophan fluorescence spectra or changes in the molar ellipticity at 222 nm as a function of the pH. After the addition of aliquots of citric acid, the pH values of the solutions containing the peptide and POPC liposomes were measured using an Orion PerHecT ROSS Combination pH Micro Electrode and an Orion Dual Star pH and ISE Benchtop Meter. Fluorescence spectra were analyzed using the Protein Fluorescence and Structural Tool Kit (PFAST)^37^ to establish the positions of λ_max_.

### Oriented Circular Dichroism (OCD) Measurements

For OCD experiments, a supported bilayer was prepared on quartz slides with special polish for far UV measurements (Starna). The procedure of cleaning the slides included the following steps: (1) soaking in a cuvette cleaner solution for 24 h, (2) rinsing with deionized distilled water, (3) sonicating for 10 min in 2-propanol, (4) sonicating in acetone, (5) sonicating in 2-propanol once again, (6) rinsing with deionized water, (7) soaking in a piranha solution consisting of 2% hydrogen peroxide and 75% sulfuric acid, and (8) rinsing with Milli-Q purified water. A POPC lipid monolayer was deposited on a quartz substrate by the Langmuir–Blodgett (LB) method using a KSV minitrough. For the LB deposition, a small amount of POPC lipids in chloroform was spread on the surface of the subphase and the solvent was allowed to evaporate for about 10 min. Next, the monolayer was compressed to 32 mN/m. When the surface pressure was stabilized, the first slide was inserted into the trough and held there for 60 s so the surface pressure would stabilize again, and then it was pulled out from the subphase with a speed of 10 mm/min. The second layer was created by fusion with POPC vesicles. About 80 μL of a state III sample (7 μM pHLIP, 0.7 mM POPC, and 2 mM pH 3–4 citrate phosphate buffer) was spread onto the slide. The process was repeated for eight more slides. The slides were then stacked on top of each other, with the spacers keeping them from sticking together, to have a complete set of 9 slides (16 bilayers). Immediately after stacking the slides, OCD spectra were measured (0 h). Afterward, the slides were kept at 100% humidity at 277 K for 6 h. At the end of 6 h, the excess solution was shaken off each slide and replaced with 80 μL of buffer at the same pH. The slides were again stacked together while filling with the buffer and stored at 100% humidity at 277 K for another 6 h. At the end of the second 6 h incubation period, the 12 h OCD spectra were measured for Arg variants in state III (the peptide inserted into the lipid bilayer of the membrane at low pH). Then, after incubating another 12 h, the 24-h OCD spectra were measured.

### Kinetics of Insertion into and Exit from the Membrane

The tryptophan fluorescence kinetics were measured using an SFM-300 mixing system (Bio-Logic Science Instruments) in combination with a MOS-450 spectrometer with temperature control set to 298 K. All samples were degassed before the measurements to minimize air bubbles in the samples. The peptide and POPC samples were incubated overnight to reach equilibrium to ensure that most of the peptide is associated with the liposome lipid bilayers. To measure the kinetics of pHLIP’s exit from the membrane, the pH of the sample was then lowered to 3.5–4.0 by adding citric acid approximately 30 min before each experiment. To follow the peptide insertion or exit, equal volumes of the peptide/POPC solution and of either citric acid or sodium phosphate dibasic, respectively, were mixed to either lower the pH from 7.2–7.4 to 3.5–4.0 or to raise the pH from 3.5–4.0 to 7.2–7.4, respectively. To monitor fluorescence intensity changes during the peptide insertion/exit induced by the pH drop or raise, the tryptophan emission signal was recorded through a cutoff 320 nm filter at an excitation of 295 nm.

### Fitting

All data was fit to the appropriate equations by nonlinear least squares curve fitting procedures employing the Levenberg–Marquardt algorithm using Origin 8.5. The pH-dependence data were normalized to a (0,1) scale and fitted with the Hill equation to determine the cooperativity (*n*) and the midpoint (p*K*) of the transition
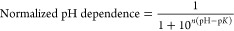
1

The kinetics data were normalized to the fluorescence intensity of state II and fitted with a multiexponential decay equation
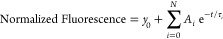
2

The value of *N* was determined by fitting with an increasing number of exponentials until the fit converged with a reduced chi-square less than 3 × 10^–5^ or until the addition of another exponential term would only lower the chi-square value by less than a factor of 10.

### System Setup and pHRE Simulations

Four pHLIP variant systems were prepared, composed of 32 amino acid residues and derived from the *wt* sequence (Table 1). The pHLIP–membrane setups were built using the previous simulations of the *wt* system^8^ as a template. In each setup, the peptide variant was generated as a full α-helical structure inserted in a 256 2-oleoyl-1-plamitoyl-*sn*-glycero-3-phosphocholine (POPC) membrane bilayer. The initial structures were built with the key aspartate (Asp13) placed in the water–membrane interface. Although *wt*-pHLIP variants, these initial structures aimed at a more unbiased approach to the system setup and equilibration protocol, since their equilibrium conformations for the inserted state may differ. After the setup, all systems were submitted to both minimization and initialization procedures, followed by a two-step equilibration protocol: the first step consisted of molecular dynamics (MD) simulations (100 ns), with the titrating residue protonation states chosen as neutral (if membrane-inserted) or charged (if solvent exposed). Additionally, distance restraints (1000 kJ/mol·nm^2^) were applied to preserve the integrity of the α-helix hydrogen bonds of every *n*^th^ – *n*^th^ + 4 residues, starting in the 17^th^ until the 28^th^ residues of the C-terminus region, which corresponds to the region located in the membrane core. The initial protonation assignment and imposed distance restraints on the helical hydrogen bonds improve the thermodynamic stability of the peptides in their relevant state III starting configuration while promoting a smoother accommodation of the surrounding lipids to the peptide presence, i.e., a decrease of nonphysical peptide–membrane configurations. The first step of the equilibration procedure using these restraints resulted in most peptides’ N-terminus segment converging to the kinked α-helical conformation, similar to what has been shown for the *wt* peptide.^14^ This behavior has also been observed recently using MD simulations and bromolipid quenching experiments (Table S1 of the Supporting Information).^20^ The second step of the protocol consisted of a 100 ns unrestrained constant-pH molecular dynamics (CpHMD) simulation at pH 6.0 to enable residue titration and remove all initial bias, equilibrating both the conformation and protonation states of the titrating residues.

**Table 1:** pHLIP Peptide Sequences of the *wt* and Arginine Variants^a^

variant	sequence
*wt*	**A**C**E**QNPIYWA**R**YA**D**WLFTTPLLLL**D**LALLV**D**A**DE**G**T**
R10	**AD**NNPFIYA**R**YA**D**LTTFPLLLL**D**LALLV**D**W**DD**
R14	**AD**NNPFIYATYA**DR**LTFPLLLL**D**LALLV**D**W**DD**
R15	**AD**NNPFIYATYA**D**L**R**TFPLLLL**D**LALLV**D**W**DD**
R17	**AD**NNPFIYATYA**D**LTF**R**PLLLL**D**LALLV**D**W**DD**

a Arginine and the titrating residues (including the termini) are highlighted in bold.

All systems were simulated using the pH replica exchange (pHRE) method.^8,38^ The pHRE is an extension to the CpHMD-L methodology^14,38−45^ that employs a replica exchange enhanced sampling technique.^46,47^ This scheme consists of a four-step cycle of *n* simultaneous CpHMD simulations (pH replicas), assigned to a pH value within a given pH range, that occurs as such: a Poisson–Boltzmann/Monte Carlo (PB/MC) calculation, followed by a molecular mechanics/molecular dynamics (MM/MD) solvent relaxation step, and then a final (MM/MD) simulation, with a pH exchange step within the previous step framework. The MC calculations assign the new protonation states using the PB-derived free energies from the system conformation of the previous cycle. The relaxation step allows the solvent molecules to accommodate the new charged states, avoiding nonphysical spikes in the system’s potential energy. The final MM/MD step samples new system conformations using the calculated protonation states. During the MM/MD step, the simulation is stopped and a pH exchange attempt occurs at a fixed frequency of 20 ps (τ_RE_), lagging 10 ps from τ_prot_, between adjacent pH replicas. If the replica exchange is accepted, according to the probability given by eq 3, the conformations and protonation states are swapped between the replicas’ pH values, thus increasing the sampling variability at both low and high energy states in every replica.
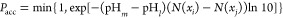
3

where pH_m_ and pH_l_ are the exchanging pH values and *x*_*i*_ and *x*_*j*_ are the number of protonated groups. For all systems, five replicates of 100 ns were performed, each replicate consisting of four pH replicas. The assigned pH values were in the 5.00 to 7.25 pH range, with a 0.75 pH step. The chosen pH range differs from previous works,^8,14^ as according to the previous equation, a smaller pH gap between replicas improves the probability and pH exchange. In these simulations, the average exchange efficiency was 40% across all systems. Each replica CpHMD cycle consisted of 20 ps (tau_prot_) steps, whereas the relaxation step was 0.2 ps (tau_rlx_). All systems were titrating the N- and C-termini and the acidic residues highlighted in Table 1. In all systems, each replicate starting conformation was obtained from the final segments of the CpHMD equilibration protocol.

### MM/MD and CpHMD Settings

All CpHMD and pHRE simulations used a modified version^40,48,49^ of the GROMACS 5.1.5 package^50^ and the GROMOS 54A7 force field,^51^ while a Python-based wrapper was used to apply the pH replica exchange method.^8,28,38^ Meanwhile, the restrained MD equilibration simulations were performed using the GROMACS 2020.1 package with the GROMOS 54A7 force field.^51^

A single cutoff scheme was applied for the treatment of nonbonded interactions. The forces were updated at every step as all pairs were under a 14 Å cutoff.^52^ Pertaining to the long-range interactions, the van der Waals forces were truncated at 14 Å, while a generalized reaction field (GRF) method, with a dielectric constant of 54^53^ and an ionic strength of 0.1 M, was used to treat the Coulombic interactions. Both peptide and lipid bond lengths were constrained using the P-LINCS algorithm^54^ and the water molecule model used was the simple point charge (SPC),^55^ whose bonds were constrained with the SETTLE algorithm.^56^ The integrator time step, for all MD simulations, was 2 fs and the conformations were sampled from an NPT ensemble. The used temperature bath scheme was the v-rescale^57^ at 310 K with a relaxation time of 0.1 ps coupled to the solute (the peptide and membrane) and solvent separately. The system pressure was kept constant with a Parrinello–Rahman barostat^58^ at 1 bar with a relaxation time of 5 ps and a compressibility of 4.5 × 10^–5^ bar^–1^.

### Poisson–Boltzmann/Monte Carlo Simulations

The Delphi V5.1 program^59^ was used to perform Poisson–Boltzmann calculations. The atom radii were obtained from the Lennard-Jones parameters of the GROMOS 54A7 force field using a 2 RT energy cutoff.^60^ The atomic partial charges were used directly from the same force field. The peptide–membrane molecular surface was defined by the following parameters: a 1.4 Å radius probe, an ion-exclusion layer of 2.0 Å, and an ionic strength of 0.1 M. The dielectric constants used were 2 and 80 for the solute and solvent, respectively. To calculate the electrostatic potential, a two-step focusing procedure was conducted with two 91 grid points. The coarse grid had a ∼1 Å spacing between the grid points, while the smaller grid had ∼0.25 Å. The defined relaxation parameters were 0.20 and 0.75 for linear and nonlinear interactions, respectively. Periodic boundary conditions for lipid bilayer systems were applied in the *x* and *y* directions. Background interaction calculations were truncated at 25 Å and the electrostatic potential convergence threshold was 0.01 kT/e.^41,42,61^

The PETIT program performed the MC calculations of the residues’ protonation states using the free energy terms obtained from the PB calculations.^62^ The proton tautomerism was accounted for all titrable groups. For each conformation, 10^5^ MC cycles were performed and each cycle corresponds to a trial change of each individual site and pairs of sites with an interaction larger than 2 p*K* units.

### Structural Characterization of the Arginine Variants

A proper configurational and local description of these peptide/membrane systems requires structural and electrostatic analytical tools. Therefore, all systems were evaluated for their electrostatic properties, such as average protonation, the p*K*_a_ of insertion (p*K*_a_^ins^),^8,14,63^ and the complete p*K*_a_ profiles of each peptide key Asp13. The most common structural characterization consists of secondary structure analysis, membrane bilayer thickness, the Asp13 membrane insertion, and its intramolecular distances to the neighboring groups. This set of analyses clarifies both the configurational and local changes between each variant peptide conformational arrangement.

In this work, the membrane insertion of the Asp13 residue was used as a guideline to discriminate other properties’ behaviors along the membrane normal. The membrane insertion of a given residue is defined by the relative difference between the average *Z* coordinates of the membrane surface reference and the residue of interest.^64^ The membrane surface reference is defined by a minimum of 10 atoms of the neighboring lipid phosphate group, within a 6 Å radius in the *xy* plane, from the residue of interest. This data can be paired to the insertion values by their time stamps, followed by a slicing procedure, using 0.5 Å insertion bins, where the pertinent data are assigned to the corresponding insertion level, hence obtaining a given property insertion profile.

We used membrane thickness calculations to quantify the local membrane deformations.^8,14^ The method performs half-thickness calculations for each monolayer within an annulus region.^64^ This region was 0.5 Å wide as it was defined within two radii centered on the peptide. Using this annulus, a scanning procedure is performed on the *xy* plane of the membrane monolayer, as both radii are simultaneously increased in a 0.5 Å step. With this approach, we describe both local deformations and membrane “bulk” (unaffected) regions (lipids usually at distances >15 Å). The presented membrane local deformations were calculated as the difference between the local half-thicknesses and the bulk region half-thickness (beyond the 15 Å cutoff). These calculations were done on all equilibrated conformation snapshots and, at the bulk regions, the thickness of both monolayers should converge to the same value, i.e., half the thickness value for a pure POPC membrane. The experimental POPC half-thickness value range was calculated by interpolating from experimental thickness measurements in the fluid range at different temperatures.^65^

To characterize the key aspartate residue microenvironment at distinct membrane media, we need to assess and identify the neighboring groups within the Asp13 first interaction shell. For each peptide variant, we calculated the number of interacting lipid phosphate and choline groups, the ArgX–Asp13 side-chain interactions, and the number of hydrogen bonds established with water molecules. All of these and other system properties were calculated as time series and as a property insertion profile. The first interaction shell cutoff value (0.52 nm) was defined from the RDF distributions for water, phosphate, and choline groups obtained from our previous work.^8^

### p*K*_a_ Profile Calculations and Electrostatic Contributions

The p*K*_a_ profiles are an important tool to assess and interpret the local electrostatics and how it affects the proton binding affinity of a pH-sensing residue. To that effect, the p*K*_a_ calculations must fulfill the following criteria: (1) each insertion bin must possess a minimum of 10 data points of each protonated state at each pH value and for each replicate; (2) in the p*K*_a_ fit procedure, all conformational sampling data must originate from at least three replicates and each replicate requires data from at least two replicas to avoid sampling bias; (3) in a titration curve, the average protonation, at a given pH value, should not be higher (by 0.05) than the average of the previous lower pH, thus ensuring monotonicity. By fulfilling these conditions, the average protonations of each pH replica are calculated and then fitted to the Hill equation to derive the p*K*_a_ values.

A semiquantitative analysis was also performed to ascertain how each electrostatic partner contributed to the p*K*_a_ of the key aspartate. This analysis required the following steps. (1) The slicing procedure of each referenced neighboring group data to the insertion profiles for all pH values. (2) Then, all pH-dependent data were used to perform a linear interpolation with the *interpolate* tool of the *scipy* module.^66^ Then, we estimated each property value for the corresponding p*K*_a_ at all insertion levels. (3) The *Random Forest Regressor* algorithm of the *scikit-learn* module^67^ was applied. A data array (90 × 5) was constructed based on the properties data of all peptide variants, including the *wt* data from a previous work.^8^ The data consisted of all values present in each electrostatic partner profile (4 independent features) and their corresponding p*K*_a_ values (dependent value). The estimator generated several classifying decision tree predictions from several subsamples of the data set obtained from a bootstrap resampling method with replacement. Then, it calculated the average of all generated outputs to improve the prediction and it determined the relative importance ranking of each feature for the model. The hyperparameters used were 2500 trees (n_estimator_) with a max_depth of 20.

### Analyses and Error Calculations

All analyses pertaining to the secondary structure, distance measurements, the number of interactions between groups of interest, and property time series were performed using the GROMACS tool package. Further analysis was performed using in-house software (http://mms.rd.ciencias.ulisboa.pt/#software) and the specified Python modules.

All p*K*_a_ error values were calculated using a Bayesian bootstrap approach. These estimations prevent fitting issues by executing 1000 bootstraps from our average protonation samples. In each bootstrap, random weights were assigned to each sample. This procedure also requires the same selection criteria (mentioned above) to obtain final p*K*_a_ and error values. For all other properties, the error bars reflect the property standard error of the mean.

## Results and Discussion

### Biophysical Characterization of the pHLIP Variants

The targeting of pHLIP peptides can be modulated using small mutations to the sequence, especially to the residues in the transmembrane (TM) region (10^th^ to 30^th^ residue).^24,68−71^ These residues dictate the thermodynamic stability of the inserted state, hence any mutation can disturb the electrostatic balance of the key titrating residues, the lipophilicity of this region, and, ultimately, the thermodynamic equilibrium of the state III peptide–membrane configurations. Cationic residues can modify the electrostatic nature of the TM region and the insertion/exit pathways;^15^ therefore, we designed four *wt* peptide variants, each with a distinct arginine position relative to key Asp13 (Table 1).

Arg residues were placed in positions 10, 14, 15, and 17 in pHLIP sequences, and they were expected to interact differently with the Asp13 residue. All pHLIP variants contained a Trp residue at the inserting end of the peptides. Changes in the fluorescence of Trp residues and peptides’ CD spectral signal were monitored during peptides’ interactions with a lipid bilayer membrane of POPC liposomes. All peptides exhibited pH-dependent pHLIP-like behavior when investigated at high and low pH values in the absence and presence of POPC liposomes (Table 2). The OCD spectra were recorded to confirm that all peptides indeed adopt the transmembrane orientation at low pH (Figure S1 of the Supporting Information). The R10 variant was investigated previously (it was called W30 in the published study).^25^ The obtained results indicate that the transition at 208–210 nm disappears in OCD spectra, proving confirmation of a transmembrane orientation of the peptides in the lipid bilayer. Also, we noted that the highest helicity in state III was observed for the R14 variant, and the lowest helical content was established for the R15 variant (Table 2 and Figure S1 of the Supporting Information).

**Table 2:** Fluorescence Parameters Obtained for Arg Variants Investigated without and with POPC Liposomes at Different pH Values^a^

variant	noPOPC λ_max_ (nm)	+POPC pH 8 λ_max_ (nm)	+POPC pH 3 λ_max_ (nm)	+POPC pH 3 helicity (mdeg)	fluor. p*K*/*n*	CD p*K*/*n*	τ_insertion_	τ_exit_
R10	352.1	350.4	339.7	–10.3	6.2/1.8	5.8/1.8	14 ms 0.2 s 5 s	42 ms 194 ms
R14	352.1	350.7	340.3	–11.9	6.0/1.1	5.8/2.6	9 ms 1.8 s 27 s	499 ms 2.3 s 14.4 s
R15	352.4	353.4	340.3	–7.5	5.9/2.8	5.6/2.6	60 ms 1.2 s 60 s	42 ms 236 ms
R17	351.3	347.5	340.3	–8.8	5.6/1.2	5.5/2.8	5 ms 0.06 s 4 s	114 ms 876 ms 8.9 s

a Position of the maximum of fluorescence spectra, λ_max_; helicity at 222 nm; mid of transition (p*K*) and cooperativity of transition (*n*) for the peptides’ partitioning into the membrane as measured by fluorescence changes, and for the coil–helix transitions as measured by CD changes; and characteristic times of insertion into the membrane, τ_insertion_, and times of exit from the membrane, τ_exit_, are shown. In solution, pHLIP forms an unstructured polymer at high pH (∼8), leading to the so-called state I. The interaction of pHLIP with the lipid bilayer of the POPC membrane at high pH (∼8) corresponds to state II. The transmembrane helical orientation of pHLIP triggered by low pH (3–5) is often called state III.

**Figure 1:**
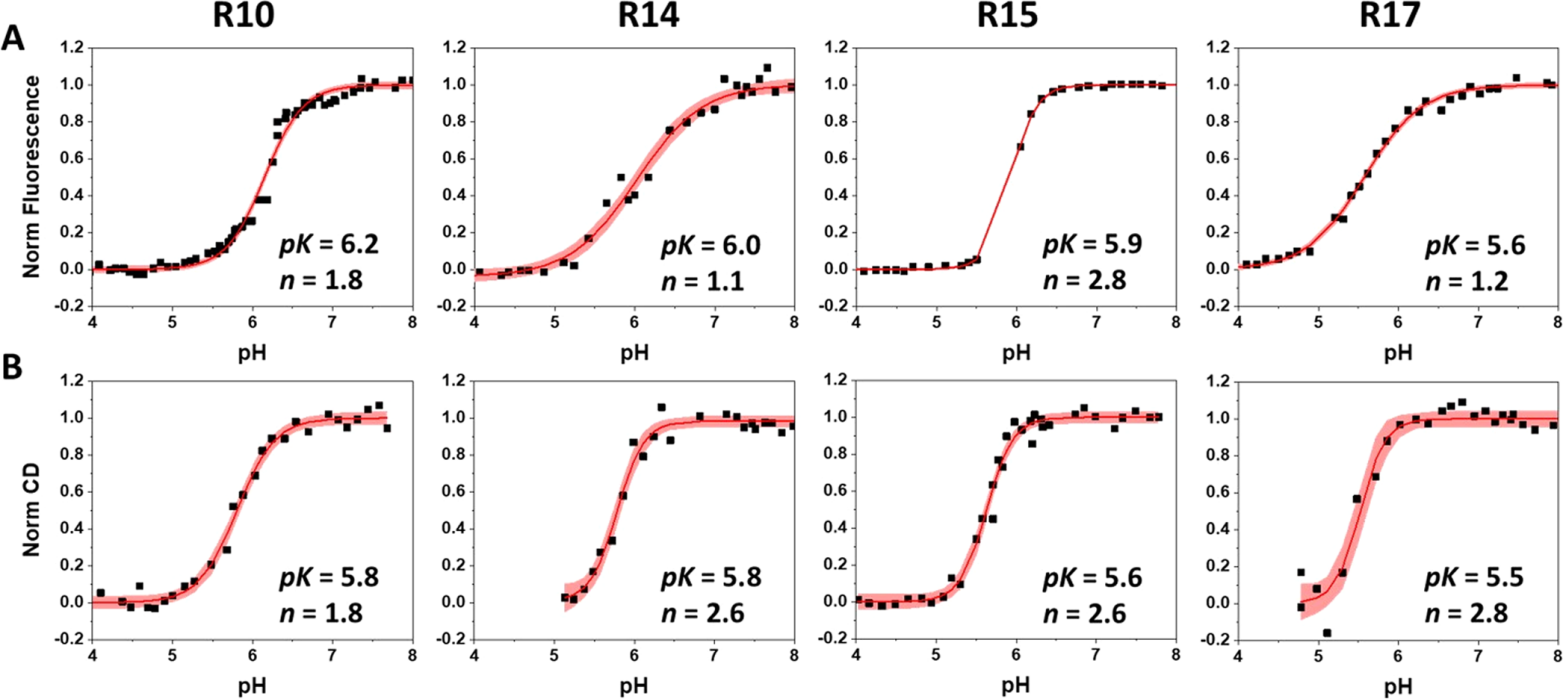
Normalized pH dependence of insertion into the membrane of POPC liposomes as determined by recording changes in the λ_max_ of tryptophan fluorescence spectra (A) and CD at 222 nm (B) are shown for the four peptide variants as a function of pH. The pink area represents the 95% confidence interval. The p*K* and *n* values are given for each peptide variant and can be found in Table 2.

Our experimental analysis of the p*K* of transition from state II (the peptide in solution at high pH in the presence of POPC liposomes) to state III (the peptide inserted into the membrane at low pH) of Arg variants revealed the trend for the reduction of p*K* moving from R10 to R17. The changes in the fluorescence signal during the transition reflect the insertion of the tryptophan residue or partitioning of the peptide into the bilayer of the membrane (Figure 1A), while the changes in the CD signal reflect the coil–helix transition (Figure 1B). The significantly lower p*K* (5.5–5.6) was established for the R17 variant compared to other peptides.

**Figure 2:**
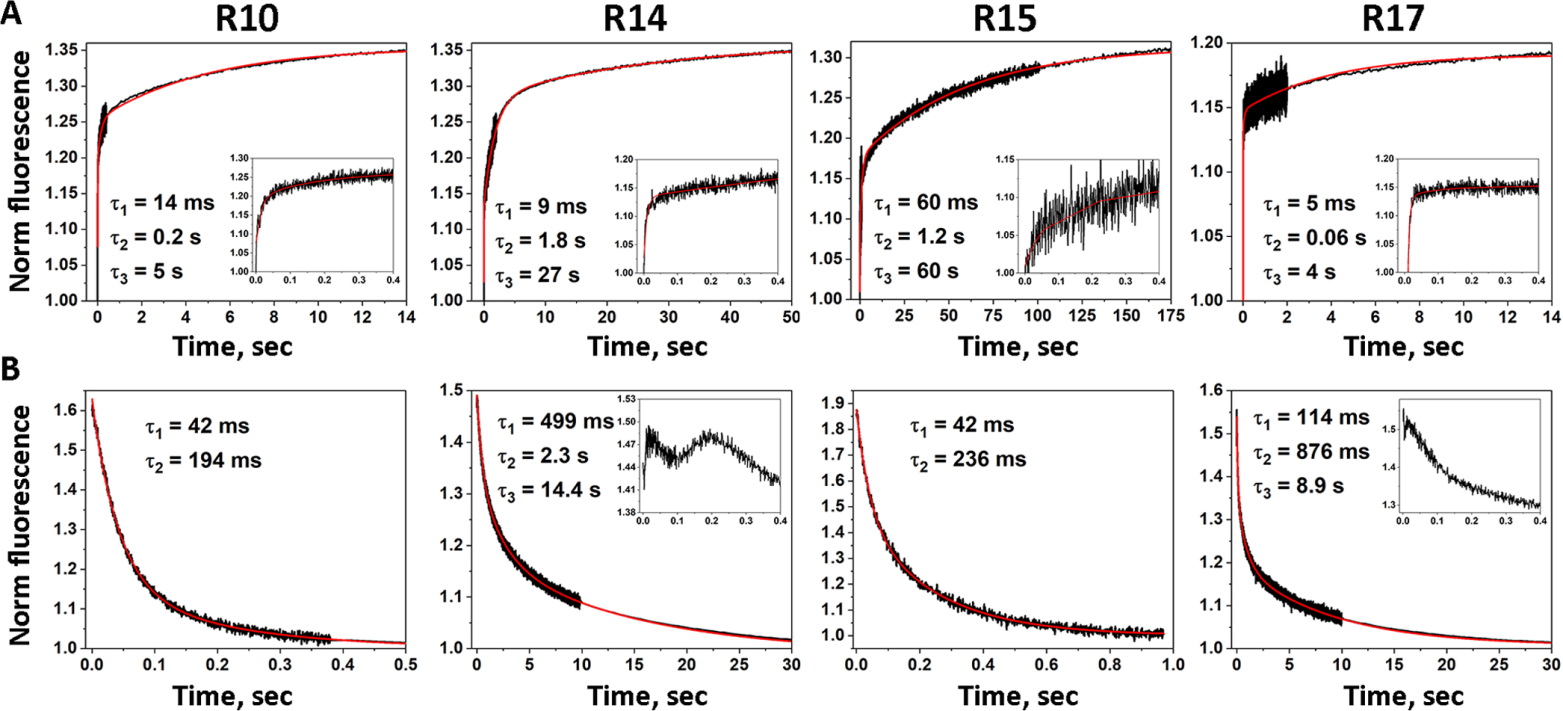
Kinetics of peptide insertion (A) into and exit (B) from the membrane of POPC liposomes monitored by changes in fluorescence as a result of drop (A) or increase (B) in pH are shown. The signals were normalized to the fluorescence in state II (the peptide and POPC at high pH). The black lines represent experimental data and the red lines the fit functions. The first 10–20 ms of insertion kinetics of all variants and the first 200 ms of exit kinetics of R14 and R17 variants were excluded from the fitting. The characteristic times are shown for each peptide variant and can be found in Table 2 of the main manuscript.

We also investigated the kinetics of peptide insertion and exit from the membrane (Figure 2). The insertion times of the different variants varied in the range of 4–60 s. The obtained data reflect the insertion and equilibration processes in the membrane. The R10 and R17 variants exhibit the fastest insertion/equilibration. The R14 variant also has a fast initial phase with a slowed final process, which was completed with a characteristic time of 27 s. The R15 variant exhibits by far the slowest insertion/equilibration kinetics compared to all variants. The exit of both the R10 and R15 variants is completed within ∼200 ms. The exit of the R14 and R17 variants is slower and was completed within 9–14 s. Both peptides showed some interesting behavior within the first 100–200 ms (insets in Figure 2B). The fluorescence intensity increases before it starts to decay in the case of the R17 variant. The R14 variant shows even two oscillations in the signal prior to the main decay. These short-time scale phenomena are not taken into account by the main decay fits for the R14 and R17 variants.

### Structural Characterization of the pHLIP Variants

**Figure 3:**
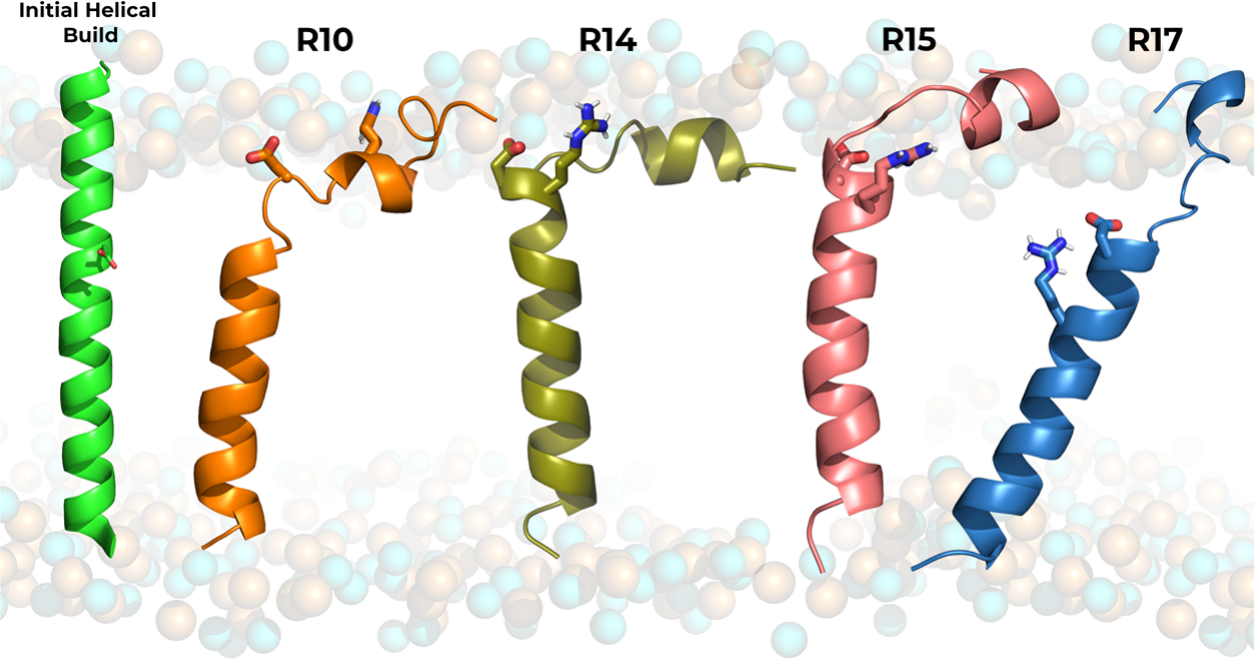
Graphical representation of the peptide conformations in the POPC membrane bilayer obtained from the CpHMD equilibration procedure. Each peptide variant (see Table 1) is shown in the cartoon with its respective color (R10–orange; R14–olive green; R15–pink; R17–blue). The unbiased full α-helical initial conformation is depicted on the left in light green. The key Asp and Arg residues are shown in sticks.

The pHRE MD simulations show that all peptides slowly converged to a similar structure, not very different from the typical *wt* α-helical conformations displaying the characteristic kink near the water–membrane interface (Figures 3 and S2 of the Supporting Information). The most important peptide and membrane properties equilibrated relatively fast, with convergence obtained after the initial 30 ns, which were discarded (Figures S3–S14 of the Supporting Information).

**Figure 4:**
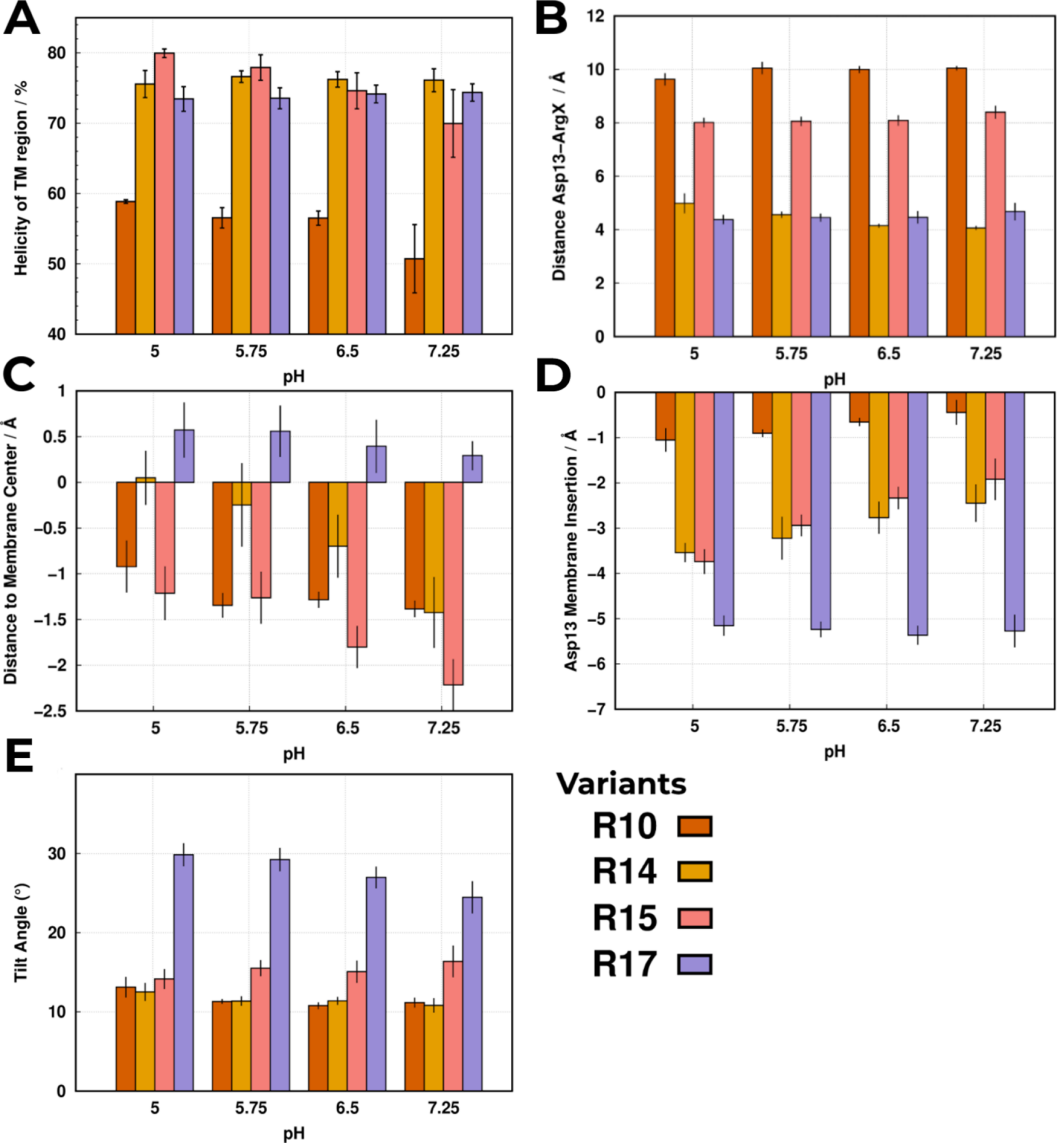
Structural analyses were performed for all variant peptides using the equilibrated segments of the pHRE simulations at all pH values. Each analysis shows the following calculation: (A) the total average helicity (α-helix) percentage of the TM region (10^th^ to 30^th^); (B) average Asp13–ArgX distance at each pH value; (C) average distance of the most central residue (Leu21) to the membrane center, where positive/negative values are closer to the outer/inner monolayer; (D) average membrane insertion of Asp13, where the negative values indicate the membrane depth; and (E) average tilt angle of the TM segment relative to the membrane normal (see Figure S15 of the Supporting Information for a schematic representation). All shown error bars reflect the standard error of the mean (SEM).

The peptides’ structural characterization highlights the unique effects of each arginine permutation on their structural stability (Figure 4A) and the local Asp13 vicinity, in particular, their specific interactions with the key Arg residues (Figure 4B). The peptide variants’ distinct folding patterns suggest that arginine mutations placed lower in the sequence (R14, R15, and R17) progressively induce larger hydrophobic mismatches (Figure 4C,D) than the *wt* sequence (Asp14 membrane insertion is −2.0 ± 0.6 Å at pH 6.0), as they increasingly expose the C-terminus hydrophobic flanking regions to the water–membrane interface, leading to more thermodynamically unstable states. More pronounced peptide tilting (Figure 4E), compared to the *wt* at pH 6.0 (16.0 ± 3.7°), and helical unfolding (Figures 4A and S2 of Supporting Information), also relative to the *wt* TM region helicity at pH 6.0 (91.5 ± 1.0%), promote internalization of the hydrophobic stretch (Pro18 to Leu24) to mitigate these mismatch effects. This is further evidenced by the progressively deeper positions (negative values) of the central Leu21 (Figure 4C), with the exception of the R17 variant where the significant peptide tilting (≈30°) counteracts the TM region vertical movement (positive values). Overall, the energy penalty associated with the internalization of the N- and C-termini charged polar residues outweighs the partial helical unfolding and structural tilting, favoring these conformational rearrangements.

Interestingly, the R10 folding pattern contrasts with the other peptides, as placing the positive guanidinium group higher in the sequence creates a TM hydrophobic mismatch in the opposite direction. The Arg10 position inverts the observed helical loss of the hydrophobic TM stretches (18^th^–21^th^ and 22^th^–24^th^) (Figure S2 of the Supporting Information) due to the closer proximity of the TM stretch to the polar environment of the outer water–membrane interface (Figure 4D). In the computational model, this proximity triggers helical loss of the hydrophobic stretch (18^th^–21^th^) to stabilize near the acyl chains (≈−1.4 at pH 5.75 in Figure 4C). However, this behavior is the opposite of what has been observed by the CD measurements (Table 2 and ref (25)), indicating that the conformational ensemble of the R10 system may not be completely representative. Nevertheless, it is clear that the arginine residue functionally works as a positive anchor that, depending on its sequence position, either propels (pulls) the peptide to (from) the hydrophobic membrane core and inner water–membrane interface region. The TM regions’ hydrophobic mismatches depend on the position permutation and strongly affect the stability of the peptide–membrane configuration, the peptide tilting, and the degree of α-helix folding in the flanking regions (Figures 4A,E and S2 of the Supporting Information).

**Figure 5:**
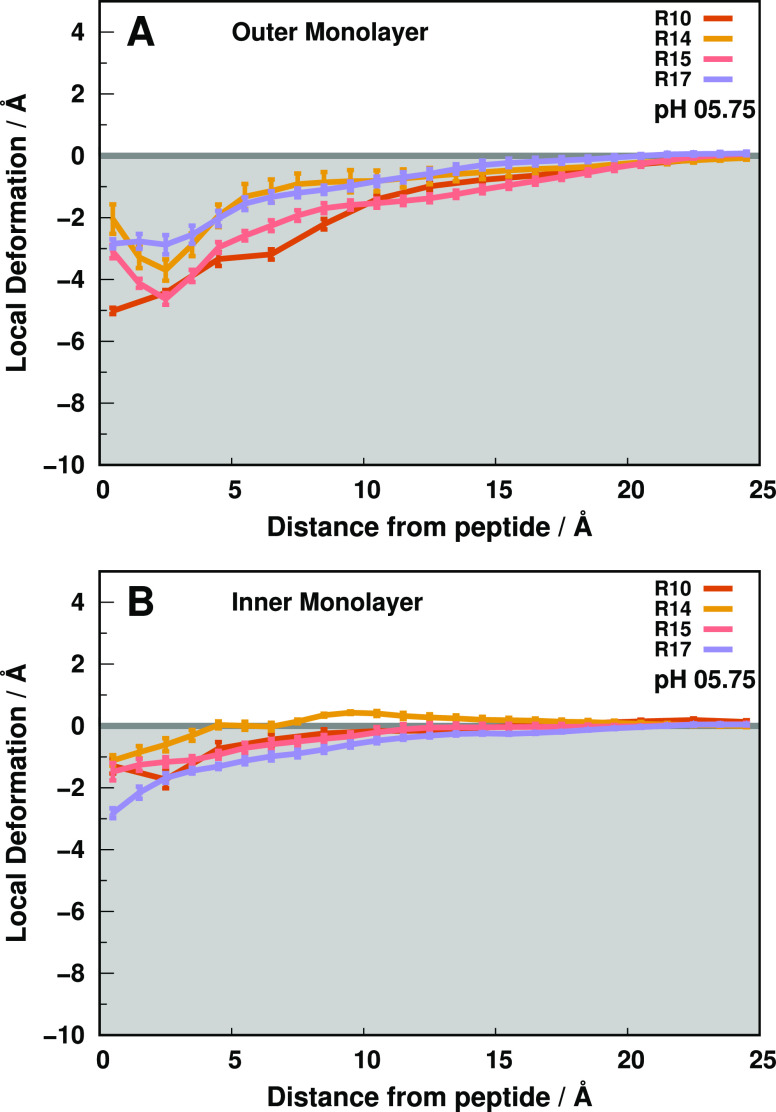
Outer (A) and inner (B) local monolayer deformation values in the *xy* plane along the peptide distance. Each colored trend represents the membrane deformations induced by one of the peptide variants at pH 5.75 until the “bulk” lipids (15 to 25 Å). The error bars represent a standard error of the mean at every 1.0 Å.

Major and minor (local) peptide movements are intertwined to define transmembrane pHLIP configurations and the local electrostatic vicinity of key Asp13. The structural disposition of the peptides imparts distinct Asp13 membrane behaviors, populating either shallow membrane regions (R10) similarly to the *wt* peptide (−2.0 ± 0.6 Å) or below the ester region (R14, R15, and R17) (Figures 4D and S16 of the Supporting Information). The internalization of a polar charged residue deeply perturbs the membrane bilayer, as water molecules and lipid headgroups typically form a stabilizing polar shell. Deeper Asp13 residues should induce larger deformations, yet our results show that the deeper R14 and R17 variants induce smaller membrane perturbations, while R15 and the more shallow R10 cause pronounced perturbations (Figures 4D and 5) correlated with a fast exit from the membrane (Figure 2 and Table 2). The decoupling between the major peptide structure and lipid bilayer deformations warrants a look at the local Asp13 environment.

Although all peptides exhibit unfolding events, the Asp13 region remains remarkably conserved throughout the simulations (Figure S2 of the Supporting Information), also observed in the *wt* peptide.^8,14^ Therefore, this behavior either preserves the lack (R10, R15), as in *wt*-pHLIP (7.5 ± 0.2 Å at pH 6.0), or the presence (R14, R17) of tight aspartate–arginine interactions (Figure 4B). The prevalent formation of salt-bridge interactions between the guanidinium and carboxylate groups occurs as the residue sequence positions (1 and 4 residues apart) place the residue side chains side-by-side or in the top/bottom positions of the α-helix (Figure S15 of the Supporting Information). Then, these salt bridges facilitate membrane embedding, despite their polar nature, as the charge neutralization decreases the need for a stabilizing solvation shell. Still, these peptides induce small deformations (∼−2 to −3 Å - R14 and R17), as transient increases in measured Asp13–ArgX distances hint at the arginine side chain breaking the salt bridge and snorkeling away to the water–membrane interface (Figures S3–S6 of the Supporting Information). This snorkeling movement tilts the TM segment at ≈15 and ≈30° for R14 and R17, respectively (Figure 4E), to minimize solvent exposure of the hydrophobic flanks, as previously noted, and induce small membrane invaginations in the inner membrane monolayer (Figure 5B). The pronounced outer membrane monolayer perturbations induced by R10 and R15 (Figure 5A) result from the membrane internalization of a well-solvated aspartate. The residue sequence positions prevent a spatial arrangement of the α-helix that favors tight ArgX interactions, hindering the aspartate stabilization through a salt bridge (Figure 4B). Consequently, choline headgroups and water molecules become the stronger interaction partners, promoting the deformation of the local lipid monolayer.

Overall, the destabilization of the water–membrane interfaces seems to mostly depend on the ability of the peptide to stabilize its Asp13 negative charge. When the Arg side chain is available to interact with Asp13 (R14 and R17), the peptides insert deeper into the membrane and minimize the water-induced deformations. Otherwise, structural constraints hamper the salt-bridge neutralization, inducing more pronounced deformations and less stable peptide–membrane configurations. Nevertheless, the arginine position is pivotal in stabilizing the peptide/membrane configuration as deeper positions experience more snorkeling events that pull the hydrophobic TM segment upward to the apolar membrane core. Altogether, the structural characterization of these peptides pinpoints an important role of the Arg position in modulating the Asp13 electrostatic environment.

### Proton Binding Affinity and Electrostatic Shell of Asp13

**Figure 6:**
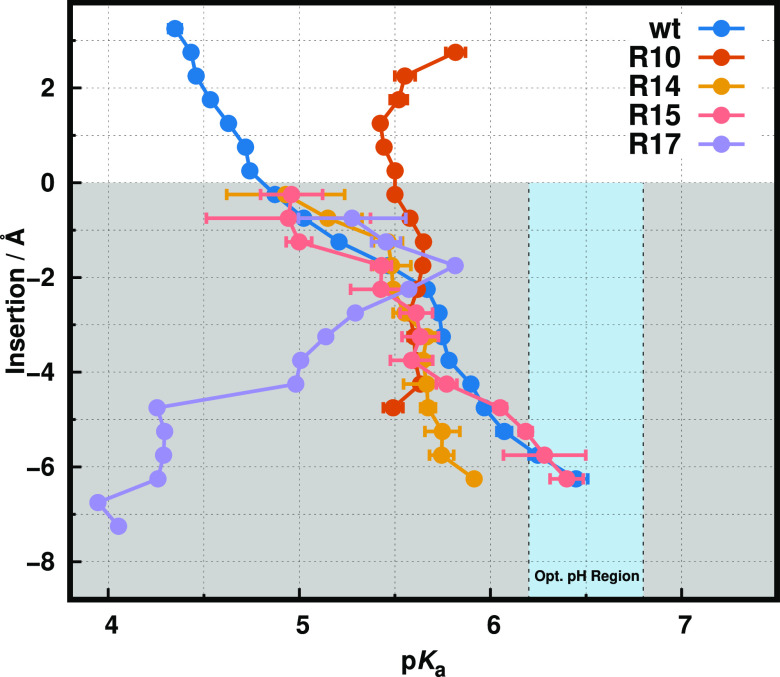
p*K*_a_ profiles of *wt*-pHLIP and its Arg variants. Each p*K*_a_ trend shows the shift along the membrane normal. The white and gray-shaded regions correspond to the water phase and membrane interior, respectively. The light blue vertical stripe identifies the pH region ideal for TME selection. The *wt*-pHLIP data were adapted from ref (8). The R10 and R17 profiles showed significant sampling limitations at deeper membrane insertion regions (<−5 Å), which resulted in the absence of data points (R10) or higher error values (more than one p*K* unit), which were omitted for clarity (R17).

The investigated peptides’ thermodynamic stability strongly depends on the (de)protonation of Asp13 to promote/hinder the insertion and exit processes. The proton binding affinity of an amino acid residue in a membrane bilayer environment is defined by the strength of the surrounding electrostatic interactions and the level of access to the solvent.^8,14,43^ Despite the complexity of depicting the different possible states of the diverse peptide–membrane configurational ecosystem, the insertion property of a residue is an indirect measurement of the peptide–membrane equilibrium, as each distinct insertion level represents a given medium (solvent, water–membrane interface, membrane core).^64^ Therefore, it is possible to accurately predict the p*K*_a_ behavior (as detailed in the p*K*a profile calculations section in the Methods section) for a given residue along the membrane normal (Figure 6).

Overall, we observe that all peptides exhibit distinct p*K*_a_ behaviors according to their own unique microenvironment. As expected, some of them exhibit a similar trend to the *wt* peptide,^8,14^ where the p*K*_a_ shifts toward higher values (Figure 6) induced by desolvation effects.^43^ However, R10 and, notably, R17 show unusual p*K*_a_ profiles, hinting at other structural and electrostatic effects to be present. The R10 profile invariance along the membrane normal and the lack of sampling in the deep membrane region (−5 to −6 Å) confirm our initial assessment that our pHRE simulations are not capturing the correct structure and protonation ensembles for this sequence. This was also hinted at by the observed disagreement in helical content between simulations and experimental data (Figure 4A). This small loss of helicity in the TM region coupled to a vertical peptide movement along the membrane normal, pulling Asp13 away from deep membrane regions, resulting in these observed prediction limitations. This could be confirmed and possibly circumvented with the use of multiple replicates in the peptide/lipid assembly/equilibration protocol, which could help identify outliers, although at a significant increase in the computational cost. All other variant peptides show good sampling and their distinct p*K*_a_ values in the deep membrane region could be calculated (Table 3).

**Table 3:** Membrane Insertion p*K* Values Obtained for the Arg Variants Using the Experimental Fluorescence Spectra and pHRE p*K*_a_ Profiles at the Deeper Membrane Regions^a^

variant	experimental	simulation
R10	6.2	—
R14	6.0	5.9
R15	5.9	6.3
R17	5.6	<5.0

a Incomplete R10 p*K*_a_ profile precluded a reliable p*K*_a_^ins^ estimation.

**Figure 7:**
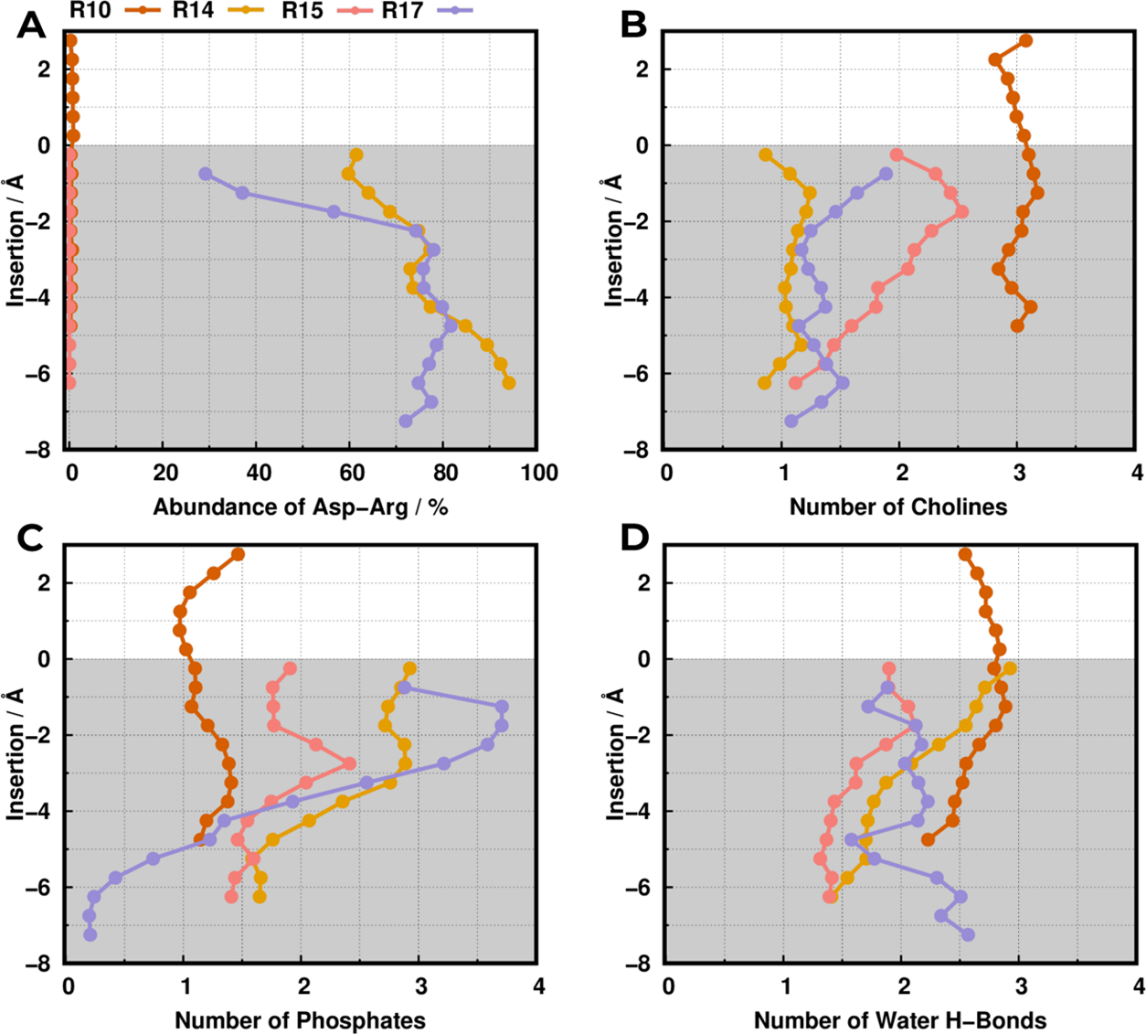
Property profiles relative to each neighboring electrostatic partner. The abundance of the ArgX–Asp13 interaction (A), the number of contacts with the phosphate groups (B), cholines (C), and water H-bonds (D) were all calculated using a distance cutoff of 0.52 nm. The white- and gray-shaded regions correspond to the water phase and membrane interior, respectively.

Although the p*K*_a_ profiles diverge in the deep membrane regions, both the structural and electrostatic analysis (Figures 4B and 7A) hint at two behavioral modalities regarding either the presence or a lack of tight arginine interactions. The lack of tight arginine interactions would indicate a certain structural similarity of the R15 profile (and R10, in principle) with the *wt*. Indeed, our predicted R15 profile (p*K*_a_^ins^ = 6.3 ± 0.1) exhibits remarkably similar behavior to the model *wt* profile (p*K*_a_^ins^ = 6.4 ± 0.1) at deep membrane regions (−5 to −6 Å), despite its small deviation from experimental insertion p*K* (p*K*^ins^ = 5.9). The omission of tight arginine interactions (Figures 4B and 7A) and a constant balance of choline and phosphate groups within the interaction shell (Figure 7B,C) further hints at analogous electrostatic environments with the *wt* peptide.^8,28^ The previously discussed structural characteristics (Figure 4A) attenuate the impact of distinct sequence positions, thus sampling equivalent peptide–membrane configurations in equilibrium.

Regarding the R14 and R17 peptides, the structural analysis highlighted tighter Asp13–ArgX interactions, hence we expect some divergence in the p*K*_a_ profiles relative to the R15 sequence. R14 shared some structural similarities with the R15 peptide, noted by only a small difference in helical content (<5%–Figure 4A) and analogous membrane monolayer perturbations (Figure 5). These resulted in similar p*K*_a_ profiles, which deviate only in the deeper membrane regions (−5 to −6 Å). The resulting small difference in their proton binding affinities stems from a rearrangement of the interaction shell, triggered by the presence of a salt-bridge interaction along the residue internalization (Figure 7A). Although the small decrease of R14 p*K*_a_^ins^ (5.9 ± 0.1), when compared to R15, can be related to its electrostatic environment, the robust experimental p*K*^ins^ (<6.0), with almost no change to R15, indicates that the arginine electrostatic contribution is probably also not decisive in R14.

The R17 peptide is an evident outlier concerning peptide behavior (Figure 6), with a very low p*K*_a_^ins^ (<5.0). The deeper regions of the p*K*_a_ profile are probably influenced by a partial lack of sampling, hinted by the large error bars (1–2 p*K* units). Nevertheless, the prominent shift to lower p*K*_a_ values upon membrane insertion is very clear and indicates an overwhelmingly positive environment that overcomes the expected desolvation effect in the apolar membrane regions. Indeed, the interaction shell is characterized by progressively more frequent (Figure 7A) and tight (Figure 4B) arginine interactions, which energetically favor the stabilization of the anionic state of Asp13. This phenomenon results from the thermodynamically stable peptide conformation (Figure 4A) that promotes side-chain interactions, precluding large solvation shells and causing smaller membrane perturbations (Figure 5). Consequently, these arginine interactions far outweigh other electrostatic contributions, as noted by the pronounced decay of phosphate interactions and a lack of competing choline and water interactions (Figure 7A,C), especially at deep membrane regions. These structural features are probably slightly overestimated in our model since the quite low proton binding affinity of this peptide has only a semiquantitative agreement with the experimental p*K*^ins^ (<5.6). Notwithstanding, it generated a structural model that helped to provide a convincing interpretation of the biophysical data.

This detailed topological discussion can also provide some insight into the membrane insertion kinetics of the different peptides (Figure 2 and Table 2). As previously established, the Arg and Asp residues can form tight intramolecular interactions in R14 and R17 (Figure 4B) since they are topologically close (Figure 3). These charge-stabilizing intramolecular interactions allow the peptides to be more amenable to membrane insertion, thus shedding the solvation shell and decreasing membrane disruption (Figure 5). In contrast, the distant Arg and Asp positions in the R10 and R15 peptides hinder these charge-stabilizing intramolecular interactions, which are replaced by anchoring intermolecular interactions with phosphate/choline groups at the membrane interface (Figure 4). The higher charge density surrounding these groups requires more water molecules (Figure 7), inflicting deeper membrane deformations (Figure 5). In sum, R14 and, especially, the R17 peptide exhibit faster membrane insertion kinetics than the R10 and R15 sequences and much slower exit kinetics compared with R10 and R15 (Table 2) as a result of their residues’ topological position.

### Which Electrostatic Interactions Drive the Asp13 p*K*_a_ Shift?

A residue p*K*_a_ derives from the delicate trade-off between the electrostatic contributions of several interacting partners within the solvation shell. Accordingly, different permutations of these effects, due to changes in the peptide microenvironment, result in distinct p*K*_a_ shifts. Nonetheless, the impact of these partner permutations is difficult to estimate, as certain neighboring interactions may have more prominent effects on the residue proton binding affinity than others. Therefore, we used a Random Forest Regressor method (see more details in the Methods section) to quantify the contributions of each electrostatic feature in the overall Asp13 p*K*_a_ values of these pHLIP variants (Table 4).

**Table 4:** Electrostatic Feature Importance Ranking Obtained for Our pHLIP Variant Peptide Models^a^

features	phosphate	arginine	choline	H-bonds	*R*^2^
coefficient	0.57	0.07	0.17	0.19	0.85

a Calculations were done using a Random Forest Regressor module, as explained in the Methods section. The *R*^2^ is the determination coefficient that evaluates the model’s predictive ability.

In our previous works,^8,14,28^ we determined that the phosphate groups along with the desolvation effect were the major factors for the anionic residues p*K*_a_ shifts. These observations are in agreement with the semiquantitative feature estimation, as the model gives a larger weight to these features (0.57 and 0.19 for phosphate groups and water hydrogen bonds, respectively). Surprisingly, the arginine contribution (0.07) seems strikingly low for the model, even though our structural data highlights how the arginine sequence position heavily shapes the electrostatic microenvironment and overall peptide stability. Although unexpected, this model only assumes direct charge contributions, hence their indirect impact in modulating the restrictive Asp13 interaction shell is not taken into account. As a result, some features may be exacerbated, such as the phosphate groups. Note that our model tries to estimate the contribution of each property within the residue interaction shell (0.52 nm), whose volume can only be occupied by a limited number of particles. When a phosphate group is tightly interacting with the aspartate, it is simultaneously shielding the aspartate from the nearby cholines as exemplified in the R14 contributions profiles (Figure 7). The spatial composition of these groups is intricately correlated to each other, leading to the information of a property change being already encoded in the others, exacerbating the estimation. Nevertheless, this analysis was still very important in weighing the importance of the group moieties that modulate the Asp13 p*K*_a_, being in qualitative agreement with our previous discussions on the key role of the phosphate groups. Overall, these results show that a thorough structural and electrostatic analysis is pivotal to obtaining a detailed picture of the molecular intricacies at play.

## Conclusions

The peptide–membrane configuration and the interactions between crucial residues modulate the delicate balance between structural and electrostatic effects. The ionic interactions between the key Asp and Arg residues define the favorable thermodynamic states, while the same configurations reorganize the local electrostatic environment sensed by the residue pair. This balance is fundamental to the acidity-dependent ability of the pHLIP peptides to interact with the membrane and their therapeutic applicability. In this work, we performed a multipronged structural characterization of pHLIP peptides with distinct arginine residue positions (R10, R14, R15, and R17), studied their impact on the proton binding affinity of key Asp13, and compared the calculations with experimental results. The pHRE simulations revealed both unique structural and electrostatic features in each arginine permutation. Overall, we observed that deeper arginine positions typically pull the aspartate away from the water–membrane interface undergoing a salt-bridge charge neutralization, although this depends on helical folding and the residues’ side chains’ topological proximity. Nevertheless, we showed that a more complex and intricate electrostatic interaction network seems to modulate the proton binding affinity across different membrane insertion environments.

In terms of the therapeutic potential of the peptide variants studied, only the R17 peptide exhibits a pH-dependence profile, confirmed by experiments (p*K*^ins^ = 5.6) and computations (p*K*_a_^ins^ < 5.0), that is markedly outside the therapeutic range (pH 6.0–6.5 at the surface of metabolically active acidic cells) and quite different from the *wt* peptide behavior. In the remaining peptide sequences, the ArgX/Asp13 direct interactions are either hindered by the peptide helical topology or outweighed by solvation. Therefore, we found that the position of the arginine group is fundamental in defining the first interaction shell of titrating Asp13. Most of the proton binding affinity contributions result from the phosphate groups’ configurational reorganization within the shell region, which is also complemented by the interactions with other electrostatic players. The arginine, when available for salt-bridge formation with key Asp, seems to act as a pH sensor inhibitor, significantly modulating the pH response of the peptide. Overall, cationic residues can be an important feature for peptide–membrane equilibria in transmembrane peptides, and, while the aspartate is the key residue that determines the therapeutic performance of each pHLIP variant, the arginine position can play a decisive supporting role in fine-tuning these clinically relevant peptides.

## Data Availability

The GROMACS package is freely available software used to perform MD simulations and can be downloaded at https://manual.gromacs.org/documentation/2020.1/download.html. PyMOL v2.5 is also free software for molecular visualization and generating high-quality images. It can be downloaded from https://pymol.org/2.

## Supporting Information Available

Comparison of membrane insertion of Trp-15 in *wt*-pHLIP in different published studies; experimental CD and OCD spectra for R14, R15, and R17 at low pH values; average helicity content per residue of peptide variants; time series data of all peptide variants at all pH values of arginine interactions with Asp13, the Leu21 distance to the membrane center, and the peptide tilt angle relative to the membrane normal; graphical representation of all peptide variants in a POPC membrane bilayer, highlighting the peptide tilt angle feature; and probability density function of Asp13 insertion for all variants at all pH values (PDF)
Starting configurations, topologies, index, and parameter files for all simulations and final conformations of all replicates at pH 6.5 (6.0 in the *wt*) (ZIP)

## Notes

O.A.A. and Y.K.R. are the founders of pHLIP, Inc. They have shares in the company, but the company did not fund any part of the work reported in this paper, which was carried out in their academic laboratories.
