# Supplementary material for: Arginine Residues Modulate the Membrane Interactions of pHLIP Peptides
Source: J Chem Inf Model. 2023 Jul 3;63(14):4433–46. doi: 10.1021/acs.jcim.3c00360 (PMC10369490; doi:10.1021/acs.jcim.3c00360)
Supplement: Supplementary file 1 — ci3c00360_si_001.pdf [file ci3c00360_si_001.pdf]

Supporting Information: Arginine Residues Modulate the Membrane Interactions of pHLIP Peptides

Tomás F. D. Silva,[†,¶] Hannah Visca,[‡,¶] Craig Klumpp,[‡] Oleg A. Andreev,[‡] Yana K. Reshetnyak,[‡] and Miguel Machuqueiro[*,†]

†*BioISI – Instituto de Biosistemas e Ciências Integrativas, Faculdade de Ciências, Universidade de Lisboa, 1749-016, Lisboa, Portugal*

‡*Physics Department, University of Rhode Island, Kingston, RI, 02881 USA*

¶*Both authors contributed equally*

E-mail: machuque@ciencias.ulisboa.pt

Phone: +351-21-7500112

Comparison of membrane insertion of Trp-15 in *wt*-pHLIP in different studies

In Ref. S1 a strong emphasis was put on the role of Ca^{2+} ions and the appearance of PS lipids in cancer and apoptotic cells to explain the ability of pHLIP to target cancer cells. We agree that ions and lipids play a role. However, recently many studies were published outlining excellent targeting of activated macrophages and fibroblasts in inflamed and cancerous tissues by pHLIP (macrophages have a very low presence of PS lipids in their membranes). pH at the surface of membranes of metabolically active cells and membrane potential, which changes with the metabolic switch of cells, play a very significant role in the pHLIP-targeted delivery of imaging and therapeutic agents in animals and humans. Moreover, cargo molecules, which are attached to pHLIP in functional studies, also contribute significantly to altering interactions with membranes. In the current study, we performed experimental measurements and calculations in the same conditions and thus can compare the results between them. Our experimental data indicate that Arg17 forming a salt bridge with Asp13 can insert deep into the lipidic phase, allowing the peptide to enter into the membrane. Although the Arg sequences in this work do not have a Trp residue nearby the 15th position, we decided to compare the insertion level of this residue in *wt*-pHLIP (data from Ref. S2) and the same residue in the "low pH" MD simulations from Ref. S1 (Table S1). Our CpHMD simulations are more realistic than the classical MD simulations performed at constant protonation. Even when starting from the most relevant protonation states, the fact that these are fixed may have influenced the peptide membrane configurations, biasing the residue insertion in the membrane to a region that may not clearly reflect a physical pH value. Nevertheless, we found a very good agreement between our 2018 data and the experimentally determined W15 bilayer depth penetration performed by bromolipid-quenching. The MD simulations of Ref. S1 seem to induce a small shift towards the water/membrane interface (higher distance from the membrane center), most likely induced by the different lipidic system or by an excess of protonation in the C-terminus residues (inducing deeper insertion

in that side and pushing the peptide outwards). In summary, we are confident that our CpHMD simulations are in agreement with most experimental data available and arguably represent the best computational model of peptides from the pHLIP family when inserted in a lipid bilayer.

Table S1: Comparison of membrane insertion of Trp-15 in *wt*-pHLIP in different studies. The Molecular Dynamics (MD) and bromolipid-quenching experimental data (Exp.) were obtained from Ref S1, while the CpHMD data was obtained from Ref. S2.

pH	Dist. Bilayer Center (Å)
MD (low pH)	$\sim$12
Exp. (low pH)	$\sim$10
4	9.1 $\pm$1.0
5	8.9 $\pm$0.8
6	8.2 $\pm$0.9
7	7.9 $\pm$0.8

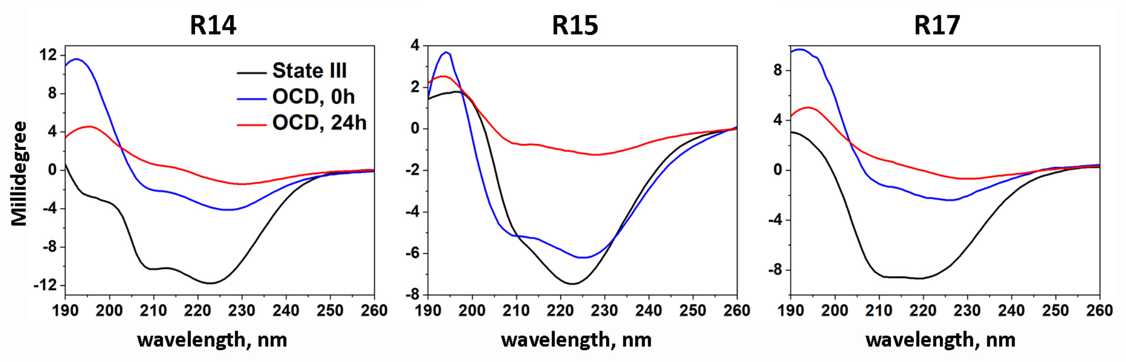

Figure S1: CD spectra recorded in solution in the presence of POPC liposomes in a cuvette at low pH (3-5) (State III), and OCD spectra recorded on supported bilayer immediately after assembling of slides (OCD, 0h) and 24 h later (OCD, 24h) at pH 3-5 for Arg variants are shown.

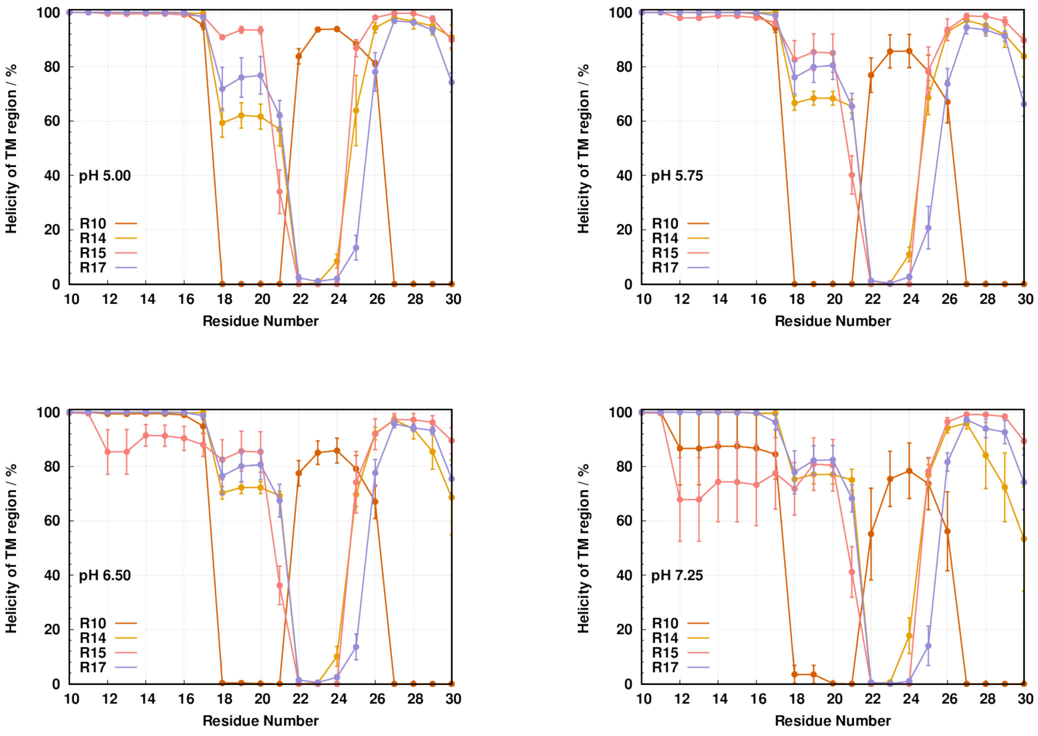

Figure S2: Average helicity (α-helix) percentage per residue number for all peptide variants, at all pH values. We focused our analysis on the transmembrane region (10th–30th residue). The error bars were obtained from the SEM.

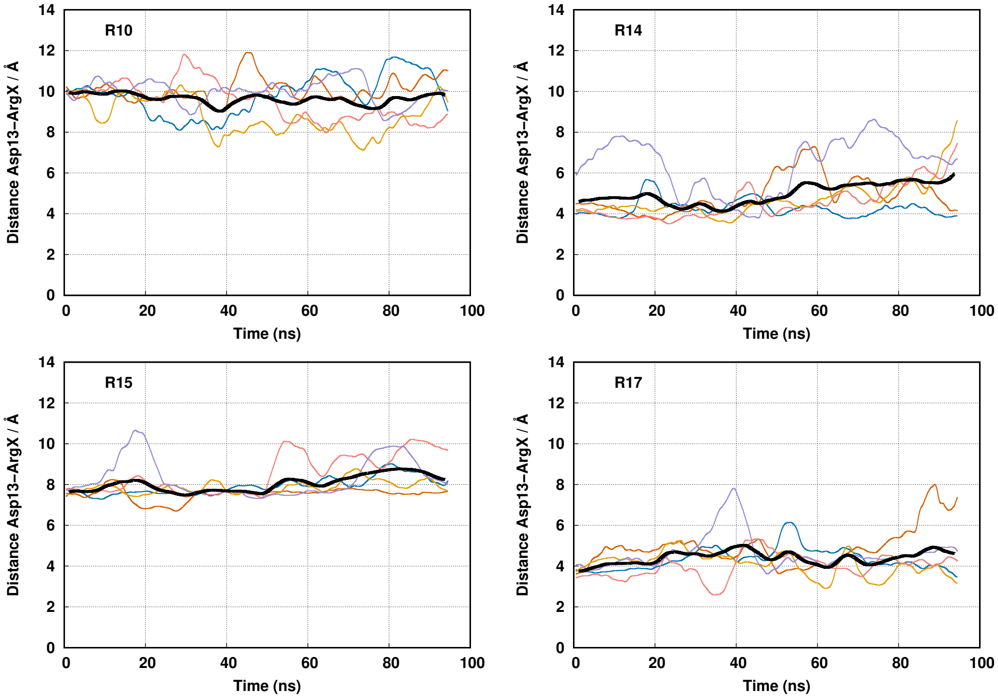

Figure S3: Time series data of the interaction distance between Asp13 and the key Arg of all peptide variants at **pH 5.00**. In each plot, the colored trends represent a different replicate, while the black trend computes the average of all replicates. All data shown was obtained from a sliding window average (bin size of 1000) to reduce the data noise.

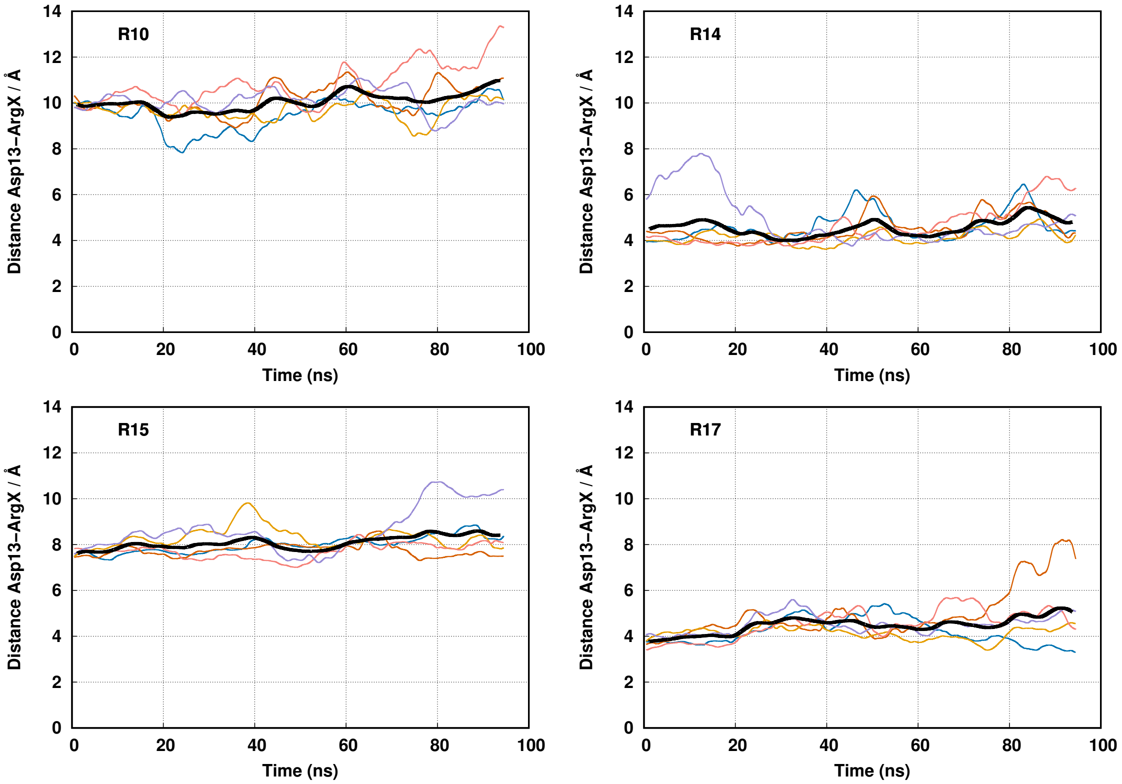

Figure S4: Time series data of the interaction distance between Asp13 and the key Arg of all peptide variants at **pH 5.75**. In each plot, the colored trends represent a different replicate, while the black trend computes the average of all replicates. All data shown was obtained from a sliding window average (bin size of 1000) to reduce the data noise.

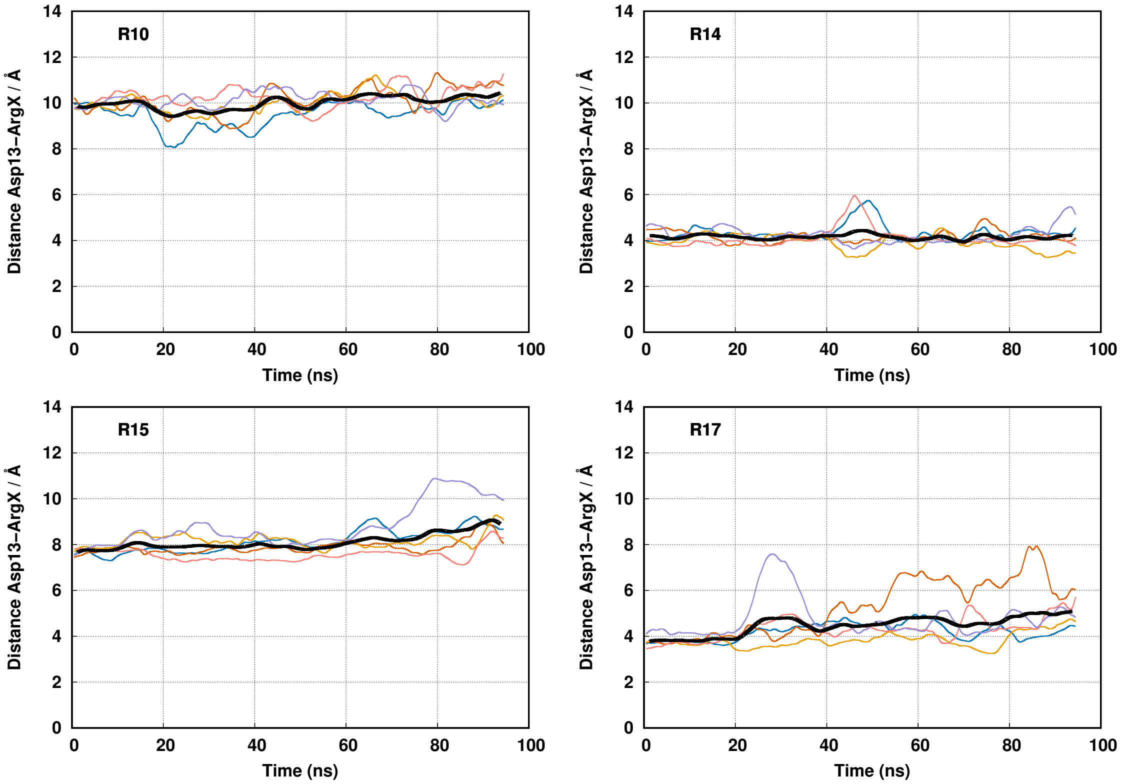

Figure S5: Time series data of the interaction distance between Asp13 and the key Arg of all peptide variants at **pH 6.50**. In each plot, the colored trends represent a different replicate, while the black trend computes the average of all replicates. All data shown was obtained from a sliding window average (bin size of 1000) to reduce the data noise.

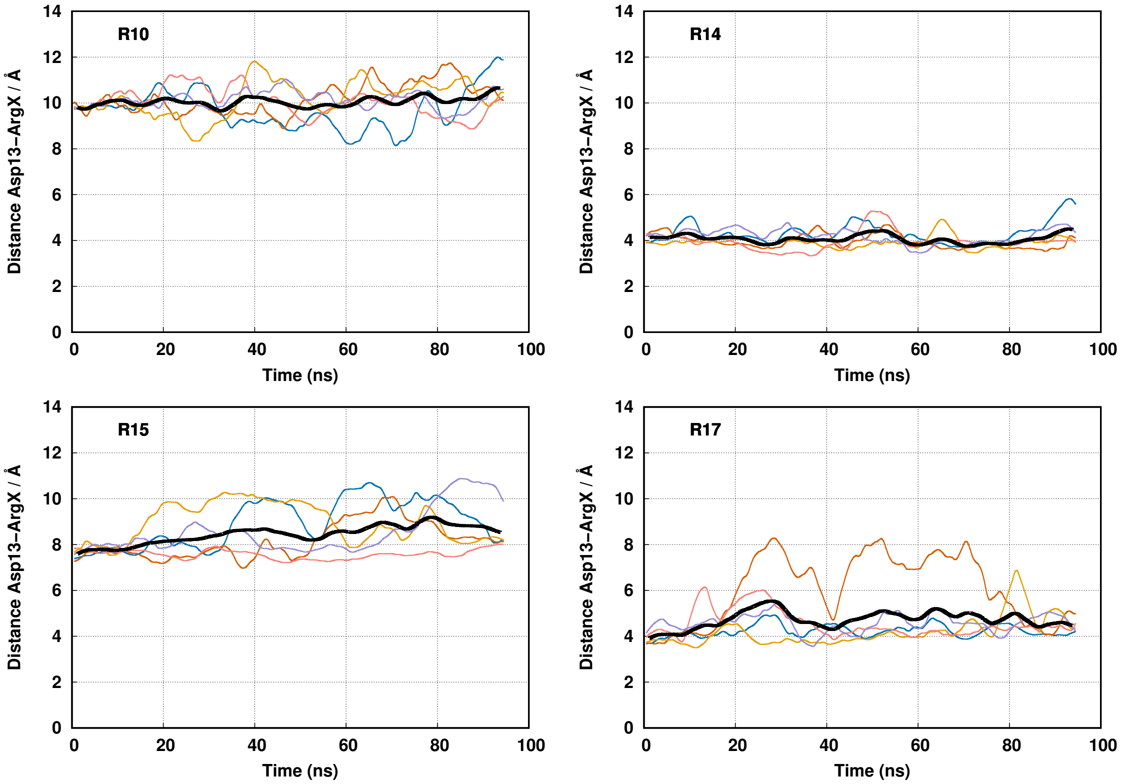

Figure S6: Time series data of the interaction distance between Asp13 and the key Arg of all peptide variants at **pH 7.25**. In each plot, the colored trends represent a different replicate, while the black trend computes the average of all replicates. All data shown was obtained from a sliding window average (bin size of 1000) to reduce the data noise.

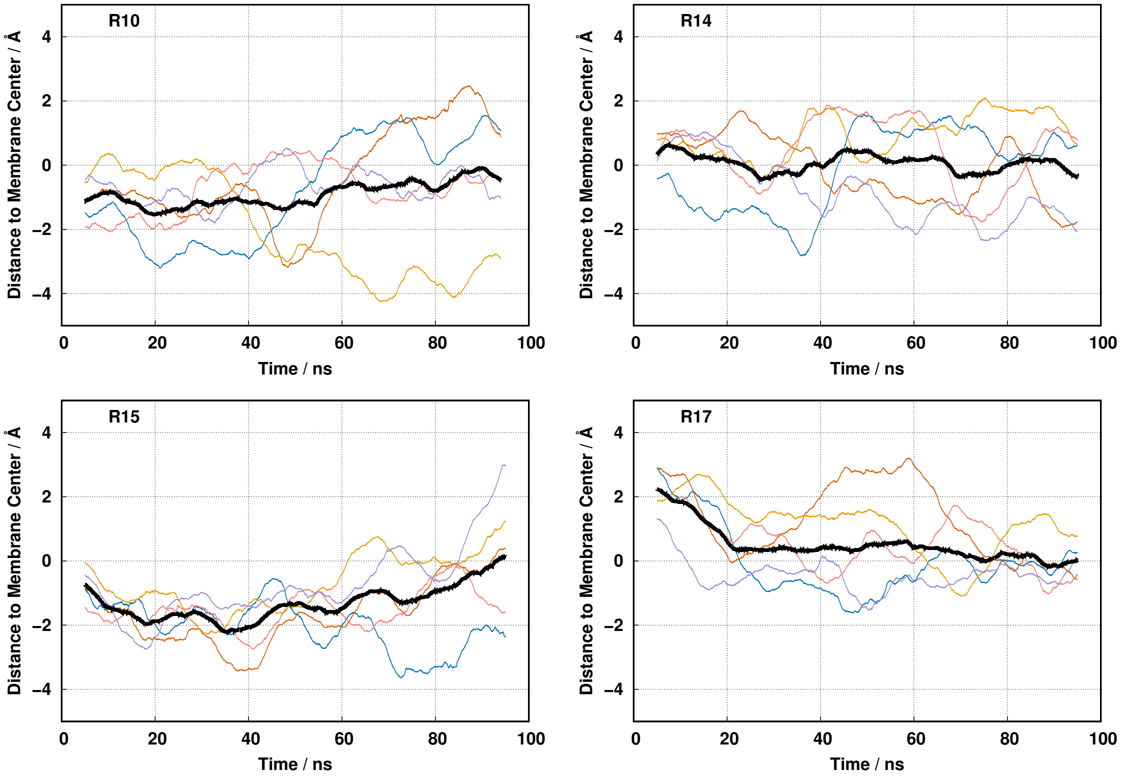

Figure S7: Time series data of the Leu21 distance to the membrane center, for all peptide variants, at **pH 5.00**. In each plot, the colored trends represent a different replicate, while the black trend computes the average of all replicates. All data shown was obtained from a sliding window average (bin size of 1000) to reduce the data noise.

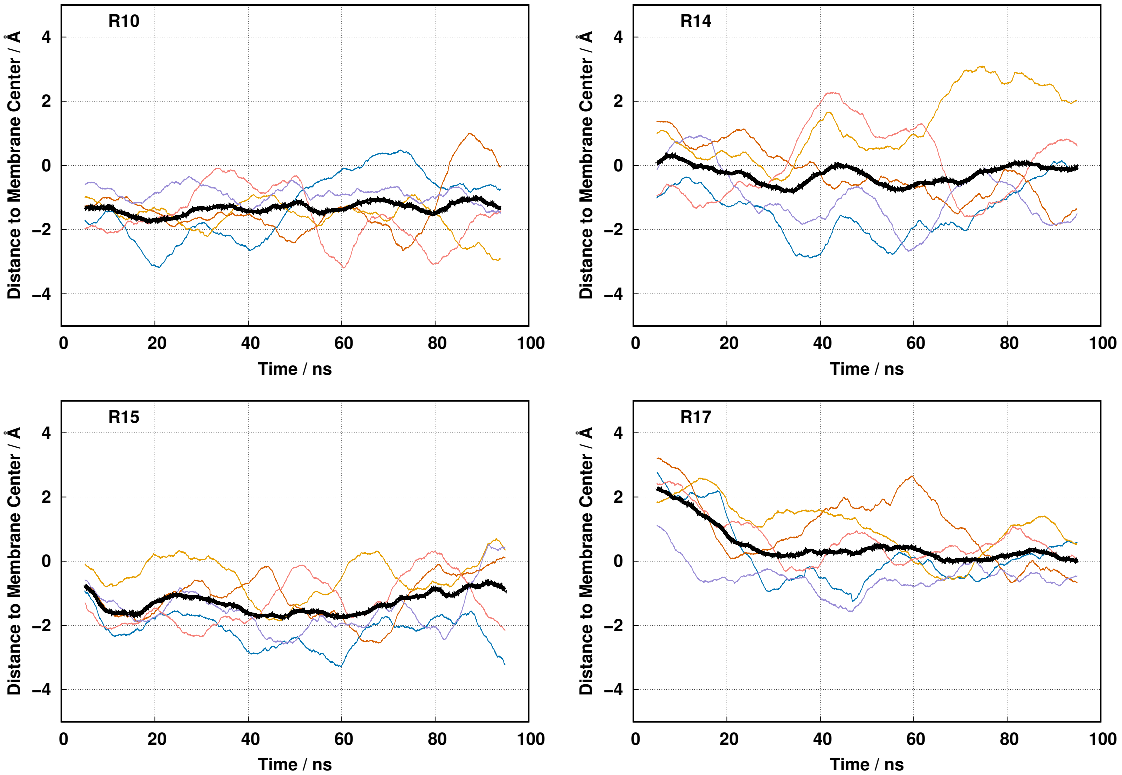

Figure S8: Time series data of the Leu21 distance to the membrane center, for all peptide variants, at **pH 5.75**. In each plot, the colored trends represent a different replicate, while the black trend computes the average of all replicates. All data shown was obtained from a sliding window average (bin size of 1000) to reduce the data noise.

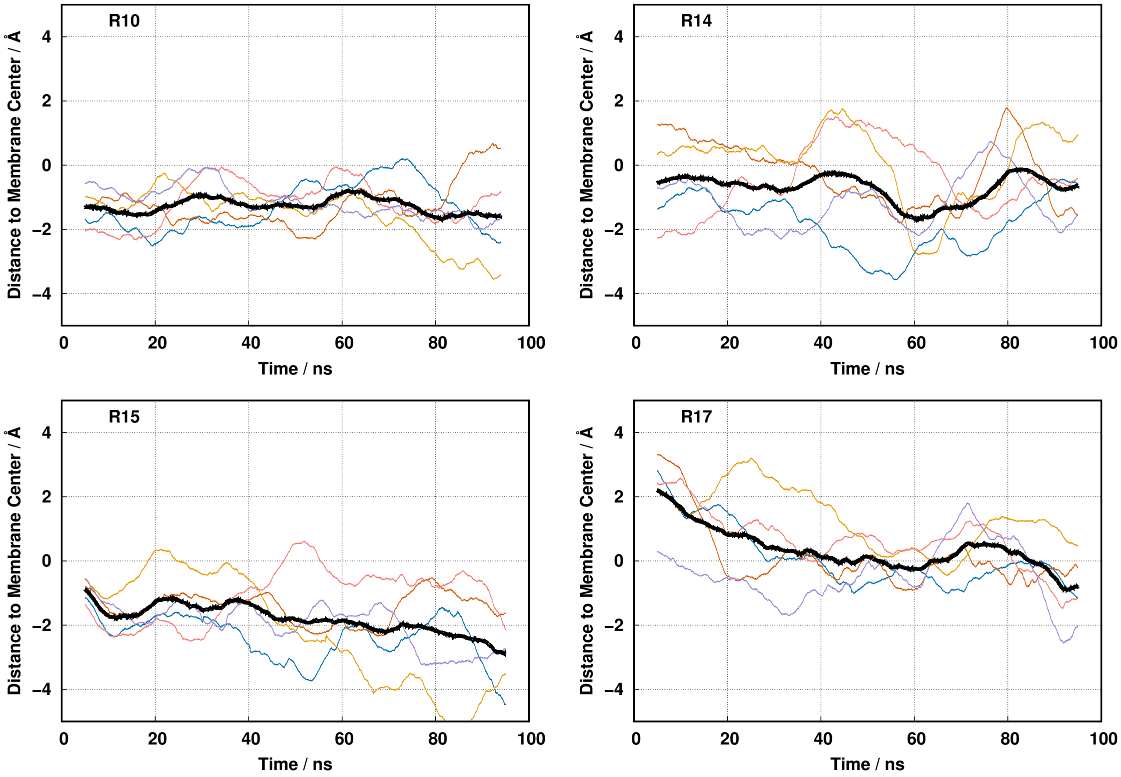

Figure S9: Time series data of the Leu21 distance from the membrane center for all peptide variants, at **pH 6.50**. In each plot, the colored trends represent a different replicate, while the black trend computes the average of all replicates. All data shown was obtained from a smoothing procedure over the original data, using a moving window average (bin size of 1000) to reduce the data noise.

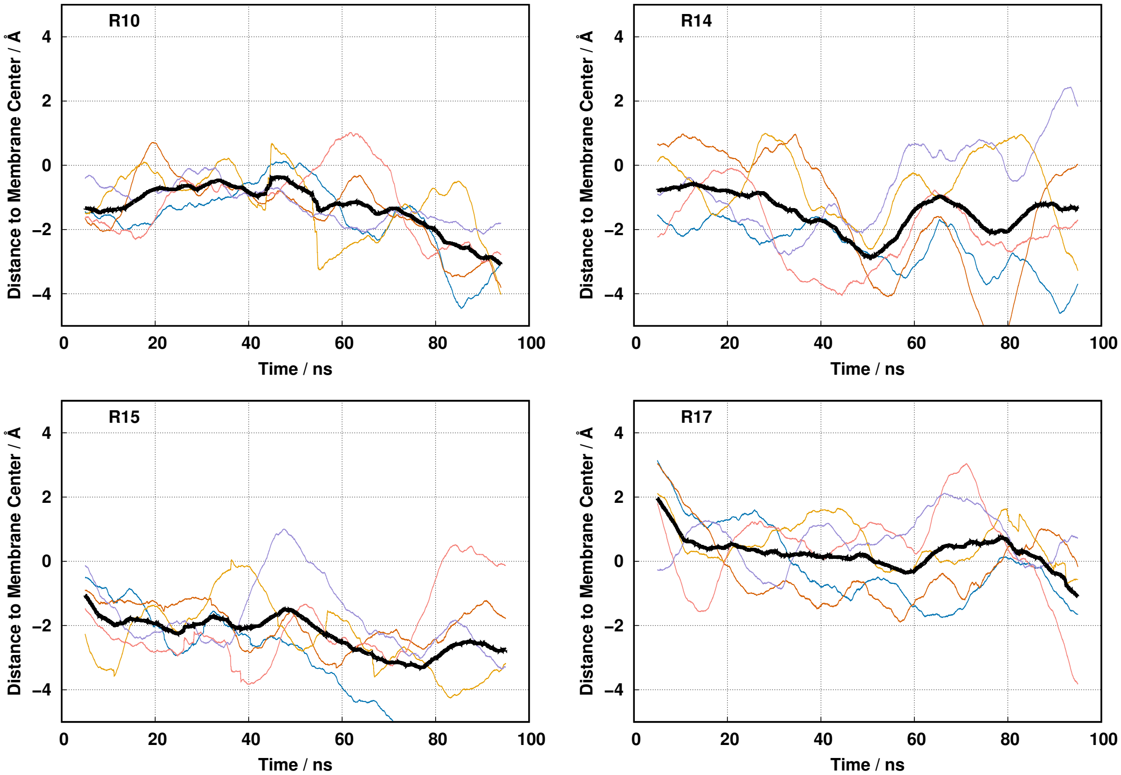

Figure S10: Time series data of the Leu21 distance from the membrane center for all peptide variants, at **pH 7.25**. In each plot, the colored trends represent a different replicate, while the black trend computes the average of all replicates. All data shown was obtained from a smoothing procedure over the original data, using a moving window average (bin size of 1000) to reduce the data noise.

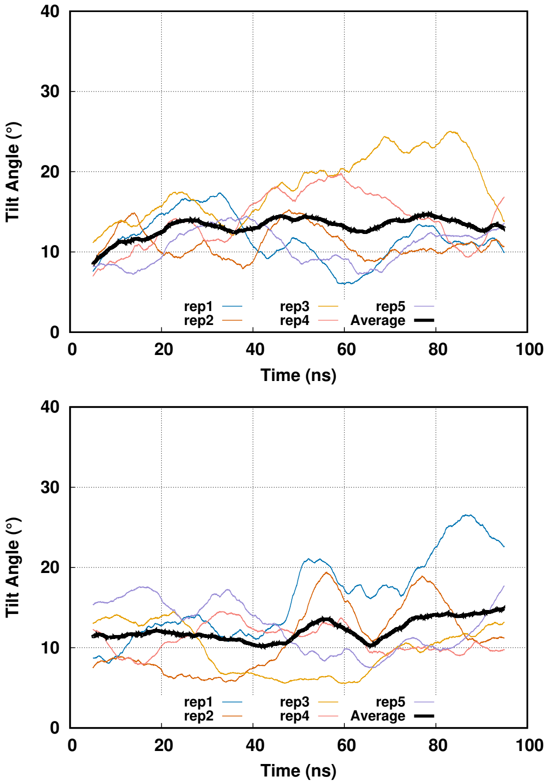

Figure S11: Time series data of the peptide tilt angle (degrees °), between the H2 (22th to 30th) helical segment and the membrane normal, of each corresponding peptide variant at a representative pH value (**pH 5.00**). The helical segment was identified as the regions with higher helical content after the central Leu21 until the C-terminus. In each plot, the colored trends represent a different replicate, while the black trend computes the average of all replicates. All data shown was obtained from a smoothing procedure over the original data, using a moving window average (bin size of 1000) to reduce the data noise.

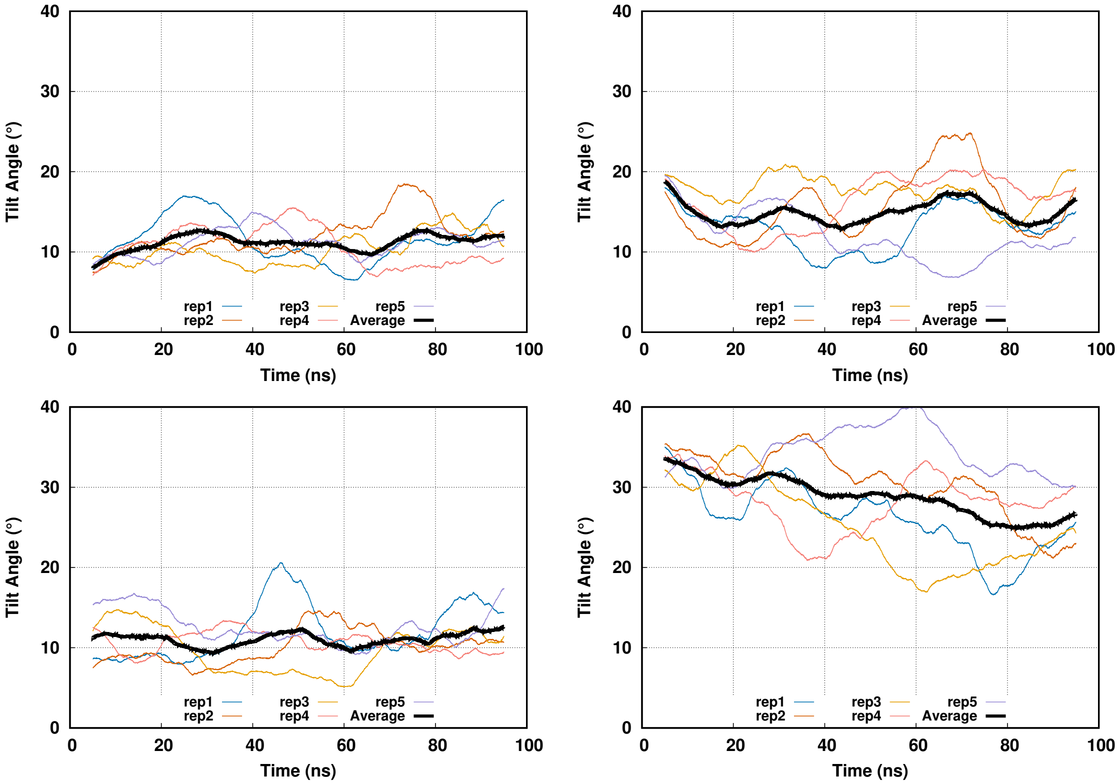

Figure S12: Time series data of the peptide tilt angle (degrees °), between the H2 (22th to 30th) helical segment and the membrane normal, of each corresponding peptide variant at a representative pH value (**pH 5.75**). The helical segment was identified as the regions with higher helical content after the central Leu21 until the C-terminus. In each plot, the colored trends represent a different replicate, while the black trend computes the average of all replicates. All data shown was obtained from a smoothing procedure over the original data, using a moving window average (bin size of 1000) to reduce the data noise.

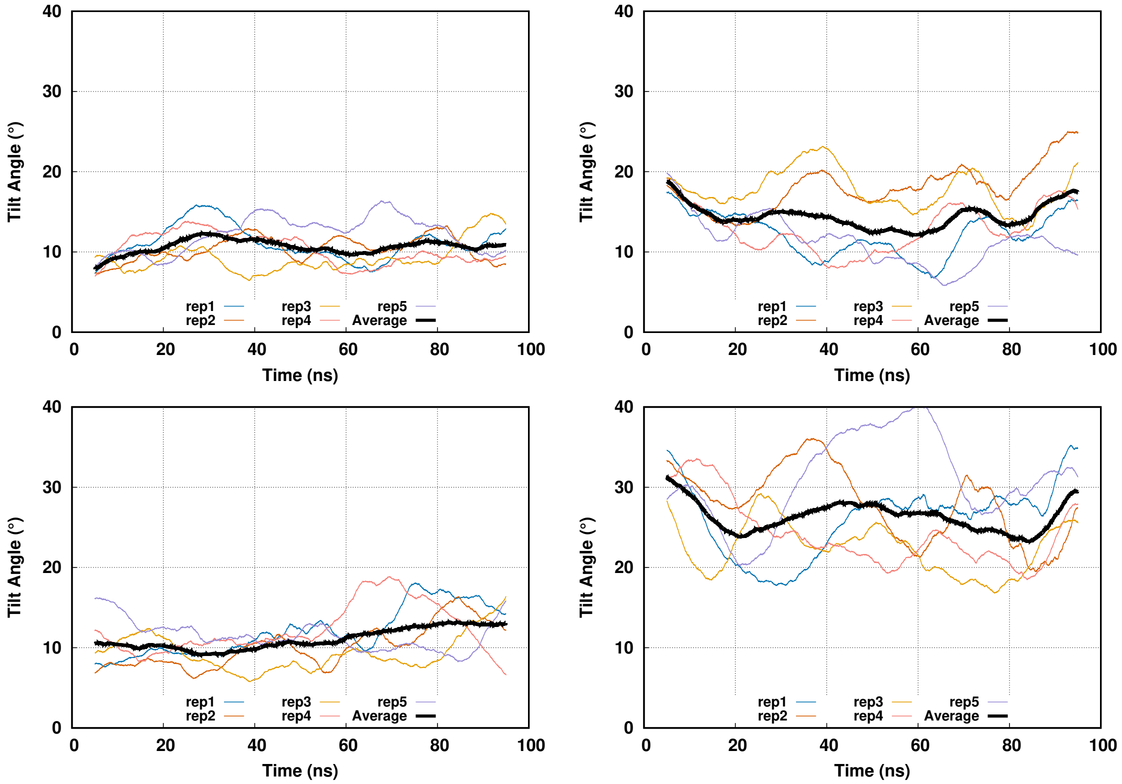

Figure S13: Time series data of the peptide tilt angle (degrees °), between the H2 (22th to 30th) helical segment and the membrane normal, of each corresponding peptide variant at a representative pH value (**pH 6.50**). The helical segment was identified as the regions with higher helical content after the central Leu21 until the C-terminus. In each plot, the colored trends represent a different replicate, while the black trend computes the average of all replicates. All data shown was obtained from a smoothing procedure over the original data, using a moving window average (bin size of 1000) to reduce the data noise.

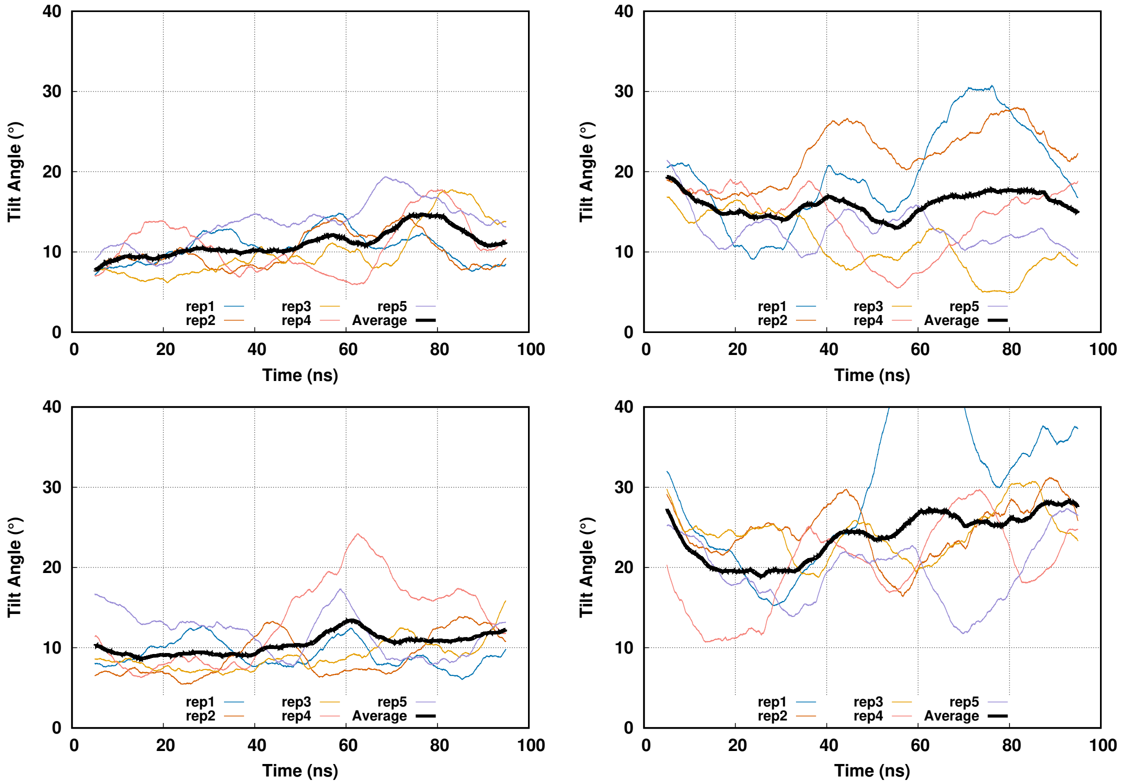

Figure S14: Time series data of the peptide tilt angle (degrees °), between the H2 (22th to 30th) helical segment and the membrane normal, of each corresponding peptide variant at a representative pH value (**pH 7.25**). The helical segment was identified as the regions with higher helical content after the central Leu21 until the C-terminus. In each plot, the colored trends represent a different replicate, while the black trend computes the average of all replicates. All data shown was obtained from a smoothing procedure over the original data, using a moving window average (bin size of 1000) to reduce the data noise.

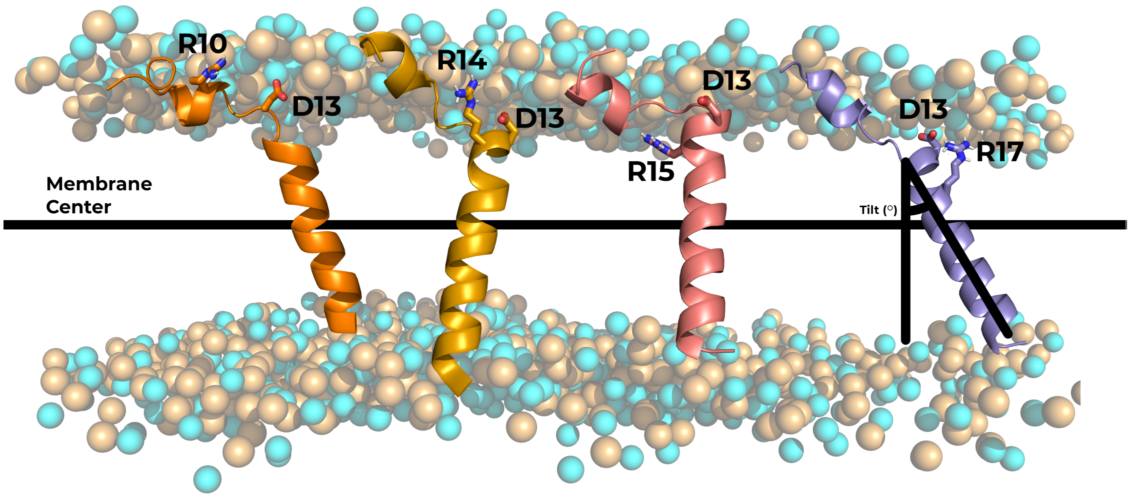

Figure S15: Graphical representation of representative peptide conformations, obtained from the final of the pHRE simulation, in the POPC membrane bilayer. The secondary structure is represented as a cartoon, while the key residues are shown in sticks. The central black line represents the membrane center and the depicted angle measures the displacement of the 2nd helix (22nd to 30th residue) relative to the membrane normal.

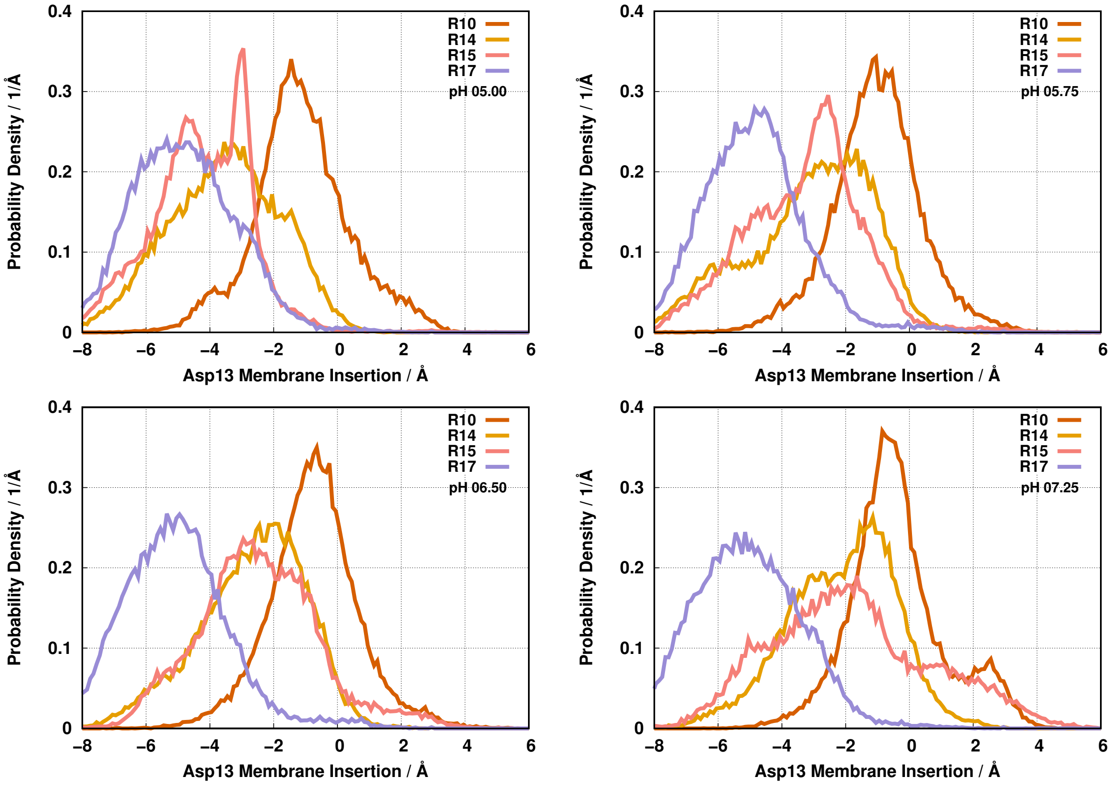

Figure S16: Probability density function of the Asp13 populating a given insertion level, at all studied pH values. Each colored trend represents a different arginine peptide variant. Negative insertion values correspond to the membrane interior.

References

(S1) Vasquez-Montes, V.; Tyagi, V.; Sikorski, E.; Kyrychenko, A.; Freites, J. A.; Thévenin, D.; Tobias, D. J.; Ladokhin, A. S. Ca2+-dependent interactions between lipids and the tumor-targeting peptide pHLIP. *Prot. Sci.* **2022**, *31*, e4385.

(S2) Vila-Viçosa, D.; Silva, T. F.; Slaybaugh, G.; Reshetnyak, Y. K.; Andreev, O. A.; Machuqueiro, M. Membrane-Induced pK_a Shifts in wt-pHLIP and Its L16H Variant. *J. Chem. Theory Comput.* **2018**, *14*, 3289–3297.
